# The Synaptic Dysregulation in Adolescent Rats Exposed to Maternal Immune Activation

**DOI:** 10.3389/fnmol.2020.555290

**Published:** 2021-01-14

**Authors:** Magdalena Cieślik, Magdalena Gassowska-Dobrowolska, Aleksandra Zawadzka, Małgorzata Frontczak-Baniewicz, Magdalena Gewartowska, Agnieszka Dominiak, Grzegorz A. Czapski, Agata Adamczyk

**Affiliations:** ^1^Department of Cellular Signalling, Mossakowski Medical Research Centre Polish Academy of Sciences, Warsaw, Poland; ^2^Electron Microscopy Platform, Mossakowski Medical Research Centre Polish Academy of Sciences, Warsaw, Poland; ^3^Department of Biochemistry and Pharmacogenomics, Faculty of Pharmacy, Medicine, Medical University of Warsaw, Warsaw, Poland

**Keywords:** neurodevelopmental disorders, maternal immune activation, lipopolysaccharide, mitochondria, oxidative stress, synaptic dysfunction

## Abstract

Maternal immune activation (MIA) is a risk factor for neurodevelopmental disorders in offspring, but the pathomechanism is largely unknown. The aim of our study was to analyse the molecular mechanisms contributing to synaptic alterations in hippocampi of adolescent rats exposed prenatally to MIA. MIA was evoked in pregnant female rats by i.p. administration of lipopolysaccharide at gestation day 9.5. Hippocampi of offspring (52–53-days-old rats) were analysed using transmission electron microscopy (TEM), qPCR and Western blotting. Moreover, mitochondrial membrane potential, activity of respiratory complexes, and changes in glutathione system were measured. It was found that MIA induced changes in hippocampi morphology, especially in the ultrastructure of synapses, including synaptic mitochondria, which were accompanied by impairment of mitochondrial electron transport chain and decreased mitochondrial membrane potential. These phenomena were in agreement with increased generation of reactive oxygen species, which was evidenced by a decreased reduced/oxidised glutathione ratio and an increased level of dichlorofluorescein (DCF) oxidation. Activation of cyclin-dependent kinase 5, and phosphorylation of glycogen synthase kinase 3β on Ser9 occurred, leading to its inhibition and, accordingly, to hypophosphorylation of microtubule associated protein tau (MAPT). Abnormal phosphorylation and dysfunction of MAPT, the manager of the neuronal cytoskeleton, harmonised with changes in synaptic proteins. In conclusion, this is the first study demonstrating widespread synaptic changes in hippocampi of adolescent offspring prenatally exposed to MIA.

## Introduction

Many environmental factors impact the brain during the key stages of prenatal development (Kern et al., 2017). Additionally, activation of the maternal immune system during pregnancy, so-called maternal immune activation (MIA), may contribute to development of several neurological complications in offspring at all stages of life. Epidemiological evidence implicates MIA as a risk factor for developing autism spectrum disorders (ASDs), schizophrenia, epilepsy, or bipolar disorder (Knuesel et al., 2014; Estes and Mcallister, 2016). It was demonstrated that infection with rubella virus, influenza, or toxoplasma gondii increases the incidence of neurodevelopmental disorders (Solek et al., 2018). Some infectious agents may evoke specific neurological complications, such as Zika virus infection causing microcephaly and other serious brain anomalies (Rasmussen et al., 2016). However, it seems that activation of the immune system and fever in general, not a specific pathogen, is responsible for early impairment of neurodevelopmental processes in offspring leading to long-term consequences in adult life. Animals exposed to MIA during pregnancy develop alterations in neuronal migration and density leading in consequence to changes in the volume of several brain structures (Bergdolt and Dunaevsky, 2019). Moreover, MIA affects dendritic structure and synaptic formation. The nature of MIA-evoked developmental changes in the central nervous system (CNS) is strictly dependent on the time of MIA (Bergdolt and Dunaevsky, 2019).

It was proposed that microglial cells that represent the immune system in the CNS play a role in development of MIA-related changes in offspring (van Den Eynde et al., 2014; Bergdolt and Dunaevsky, 2019). Microglia is characterised by its high versatility in reacting to changes in the microenvironment. Microglial cells adopt different phenotypes in response to different stimuli (phenotype polarisation). “Classic” phenotype M1 (often referred to as cytotoxic), as a reaction to infection or brain tissue damage, is characterised by production of pro-inflammatory cytokines, matrix metalloproteinases, and free radicals. Microglia M1 is well-prepared to effectively kill microorganisms but may be also harmful to neurons when overactivated. “Alternative” phenotype M2 (referred to as neuroprotective) is typical of microglial cells acting as anti-inflammatory and neuroprotective. Transcriptomic studies suggest a broad spectrum of microglia phenotypes *in vivo*, wherein M1 and M2 are only two possible options (Xue et al., 2014). The role of microglia in neurodevelopmental alterations after MIA was studied in several experimental rodent models using mainly two common immunostimulants: lipopolysaccharide (LPS) and polyinosinic-polycytidylic acid (PIC, also abbreviated as poly [I:C]). LPS is a component of the wall of gram-negative bacteria, and PIC is a synthetic analogue of dsRNA (mimetic of viral dsRNA). Although both LPS and PIC activate innate mechanisms of immune response by stimulation of Toll-like receptors (Tlr4 and Tlr3, respectively), the response differs significantly (Konat, 2016). The mechanisms responsible for synaptic alterations in the adult brain evoked by LPS-related MIA are hardly understood. Therefore, in our experiments, we used a rat model of MIA, based on systemic administration of LPS at a relatively low dose (100 μg/kg of body weight) to pregnant females at day 9.5, which in humans corresponds to the early/middle foetal life (Kirsten et al., 2010a). The growing body of evidence indicates that activation of the mother's immune system in this phase of embryonic life may significantly impact development with prolonged effects, but the underlying mechanism is unclear (Kirsten et al., 2010b, 2013; Hornig et al., 2018; Bergdolt and Dunaevsky, 2019; Cieślik et al., 2020).

The aim of the current study was to clarify the mechanism responsible for synaptic dysregulation in hippocampi of offspring exposed to MIA in prenatal life. We focused on the hippocampus, which was implicated in autism. It was shown that the hippocampus is especially sensitive to inflammatory insults in both early pregnant and adult life, which may be a part of the pathogenesis of neurodevelopmental disorders, including autism. Our results demonstrated widespread synaptic changes in hippocampi of adolescent offspring prenatally exposed to MIA. Remarkably, despite an increased level of glial markers, Iba1 and s100β, proinflammatory signalling was not upregulated in hippocampi of MIA rats.

## Materials and Methods

### Chemicals

LPS (from *E. coli* serotype 055:B5; Lot 113M4068V; toxicity 3 × 10^6^ U/mg), TRI reagent, DNAse I and bovine serum albumin (BSA) were from Sigma-Aldrich (St. Louis, MO, USA). Goat polyclonal anti-Iba1 antibody and mouse monoclonal anti-MTCO1 antibody were from Abcam (Cambridge, UK). Donkey anti-goat IgG antibody, mouse monoclonal anti-synaptophysin antibody, mouse monoclonal anti-synapsin antibody, rabbit polyclonal anti-phospho-synapsin (Ser62/67) antibody, mouse monoclonal anti-PSD-95 antibody, mouse monoclonal anti-VAMP1/2 antibody, mouse monoclonal anti-MAPT antibody, rabbit polyclonal anti-Cdk5 antibody, rabbit polyclonal anti-p35/p25 antibody, mouse monoclonal anti-nitrotyrosine antibody, mouse monoclonal anti-arginase antibody, rabbit polyclonal anti-iNOS antibody, mouse monoclonal anti-ND1 antibody, and mouse monoclonal anti-syntaxin-1 were obtained from Santa Cruz Biotechnology Inc. (Santa Cruz, CA, USA). Rabbit polyclonal anti-synaptotagmin-1 antibody, rabbit monoclonal anti-phospho-MAPT (Ser416) antibody, mouse monoclonal anti-phospho-MAPT (Ser396) antibody, rabbit monoclonal anti-Gsk-3β antibody, rabbit polyclonal anti-phospho-Gsk-3β (Ser9) antibody, rabbit monoclonal anti-SNAP25 antibody, rabbit monoclonal anti-mTOR antibody, rabbit monoclonal anti-phospho-mTOR (Ser2448) antibody, rabbit monoclonal anti-p38 antibody, rabbit monoclonal anti phospho-p38 (Thr180/Tyr182) antibody, and rabbit polyclonal anti-vinculin antibody were obtained from Cell Signalling (Beverly, MA, USA). Rabbit polyclonal anti-Gsk-3β (Ser389) antibody was from Proteintech Group, Inc. (Rosemont, IL, USA). Rabbit polyclonal anti-GAPDH antibody, rabbit polyclonal anti-phospho-MAPT (Ser199/202) antibody, goat anti-rabbit IgG antibody and mouse monoclonal anti-s100b antibody were from Sigma-Aldrich (St. Louis, MO, USA). Sheep anti-mouse IgG antibody was from GE Healthcare (Little Chalfont, Buckinghamshire, UK). Antibodies for immunohistochemistry: rabbit polyclonal anti-IL-1 beta, rabbit polyclonal anti-liver arginase, goat polyclonal anti-Iba1, and goat polyclonal anti-GFAP were from Abcam; donkey polyclonal anti-Goat (Alexa Fluor 594) and donkey polyclonal anti-Rabbit (Alexa Fluor 488) were from Thermo Fisher Scientific (Paisley, UK). Chemiluminescent reagent Clarity Western ECL Substrate and Bio-Plex Pro Rat Cytokine 23-Plex Assay were from Bio-Rad Laboratories (Hercules, CA, USA). Protease inhibitors cocktail cOmplete was from Roche Diagnostics GmbH (Mannheim, Germany). Glutathione Assay Kit and 2′,7′-dichlorodihydrofluorescein diacetate (DCFH-DA) were from Cayman Chemical Company (Ann Arbour, MI, USA). Complex I Enzyme Activity Microplate Assay Kit and Complex IV Rodent Enzyme Activity Microplate Assay Kit were from Abcam (Cambridge, UK). Mitochondria Isolation Kit was from Sigma-Aldrich (St. Louis, MO, USA). Reagents for reverse transcription (High Capacity cDNA Reverse Transcription Kit) and PCR (TaqMan Fast Advanced Master Mix) were from Thermo Fisher Scientific (Paisley, UK). Micro RNA purification kit was from A&A Biotechnology (Gdynia, Poland). DMSO and all other common reagents were from Sigma-Aldrich (St. Louis, MO, USA). The full list of the chemicals, antibodies etc. is presented in Supplementary Materials.

### Animals

Experimental MIA was induced in female 2- to 3-month-old (200–250 g) Wistar rats, supplied by Animal House of Mossakowski Medical Research Centre PAS (Warsaw, Poland) which operates breeding of small rodents with the SPF standard. The animals were maintained under controlled temperature and humidity conditions on a 12-h light/dark cycle. All of the experiments conducted on animals were approved by the Local Ethics Committee for Animal Experimentation in Warsaw (reference numbers 4/2014, 60/2015, 64/2015, 361/2017 WAW2/083/2018, and WAW2/148/2018) and were carried out in accordance with the EU Directive 2010/63/EU for animal experiments. Every effort was made to minimise the number of animals used and reduce the amount of pain and distress.

### Experimental Procedure

The rat pregnancies were achieved by housing a male and a female overnight. The next morning, female rats were separated, and the mid-day of that day was defined as gestation day 0.5 (GD0.5). The pregnant female rats were identified and MIA was evoked by intraperitoneal (i.p.) administration of LPS from *Escherichia coli* (Sigma-Aldrich, Saint Louis, MO, USA; serotype 055:B5) (100 μg/kg b.w.) at GD9.5 (Kirsten et al., 2012; Cieślik et al., 2020). Females from the control group received i.p. administration of analogous volume of vehicle (sterile 0.9% NaCl). Maternal sickness behaviour was monitored for 24 h from LPS administration. All dams were allowed to give birth and nurture offspring under normal conditions. The day of birth was recorded as postnatal day (PND) 1. On PND 7 each litter was equalised (random selection) and the number of pups was limited to 10 (both male and female). On PND 22–23, rat pups were separated and housed in groups of 3 or 4 in open polycarbonate cages in an enriched environment. To avoid the interference of the hormonal disturbances/changes only males were selected for further experimental procedures. To reduce the risk of litter effect animals from at least 3 L in each experimental group (random selection) were tested. A panel of behavioural tests was applied to characterise some ASD-related changes (Cieślik et al., 2020). Offspring male rats from the control and MIA groups were analysed at PND52-53. After decapitation, brains were quickly removed, and hippocampi were dissected on ice.

### Transmission Electron Microscopy (TEM) Analysis

Rats were anaesthetized with a ketamine/xylazine combination (100 mg/kg b.w. for ketamine and 10 mg/kg b.w. for xylazine) and perfused through the ascending aorta initially with 0.9% NaCl in 0.1 M sodium-potassium phosphate buffer (PBS), pH 7.4, and after with 2% paraformaldehyde and 2.5% glutaraldehyde in 0.1 M cacodylate buffer, pH 7.4 at 20°C. Material for ultrastructural studies was sampled from the hippocampi (cornu ammonis, subregions CA2 and CA3) of all rat groups. Specimens were fixed in the ice-cold fixative solution for 20 h and placed in a mixture of 1% OsO_4_ and 0.8% K_4_[Fe(CN)_6_]. After dehydration in a series of ethanol gradients, hippocampus samples were embedded in epoxy resin (Epon 812). Ultra-thin sections (60 nm) were examined by TEM (JEM-1200EX, Jeol, Japan). To assess the dimensions of synaptic vesicles in nerve terminals, the outermost thickness of each vesicle was taken as its diameter. Microphotographs from each animal were randomly taken and analysed until the number of counted SV reached 150. All visible vesicles on all selected microphotographs were counted/measured. The mean value from each animal was used in calculation. The number of animals in each group is indicated by “n” number.

### Immunohistochemistry

Rats were anaesthetized with a ketamine/xylazine combination (100 mg/kg b.w. for ketamine and 10 mg/kg b.w. for xylazine) and perfused through the ascending aorta initially with 0.9% NaCl in 0.1 M PBS, pH 7.4, and after with 4% paraformaldehyde. Brains were removed and post fixed for 3 h at 4°C in the same fixative solution. Following post fixation, brains were cryoprotected overnight in 20% sucrose solution in 0.1 M PBS, frozen on dry ice and stored at −80°C. Next, the brains were delivered on dry ice and cooled in a cryostat (Leica CM1850) for 45 min at −27°C. Frozen brains were fastened to the table in the cryostat using tissue freezing medium and coronally sectioned at 40 μm thickness. Analysis was performed in cornu ammonis, subregions CA2 and CA3 region of hippocampus (bregma −3.00 to −3.36 mm) identified according to the Rat Brain Stereotaxic Atlas (Paxinos and Watson, 2007). The free-floating sections were washed 3 times with 0.1 M PBS for 5 min and incubated in 1% hydrogen peroxide in 0.1 M PBS for 30 min to inhibit/quench endogenous peroxidases. After washing with 0.1 M PBS (3 × 5 min), slices were incubated in blocking solution (5% Normal Donkey Serum in 0.1 M PBS + 0.3% TritonX100) for 1 h at room temperature (RT). The incubation with primary antibodies was performed in 5% NDS, 1% BSA, 0.3% TritonX100, and 0.1 M PBS for 1 h at RT and overnight at 4°C. The next day, the sections were washed with 0.1 M PBS (3 × 5 min), incubated in dark with fluorescently labelled secondary antibody in 5% NDS, 1% BSA, 0.3% TritonX100, and 0.1 M PBS for 1 h at RT, and washed with 0.1 M PBS (3 × 5 min). The sections were then mounted onto glass slides, air dried, and coverslipped with ProLong Gold Antifade Mountant with DAPI. Negative controls were performed with the same procedure omitting the primary antibodies. IHC results were examined using a confocal laser-scanning microscope, Zeiss LSM 780/ELYRA PS.1. (Carl Zeiss Meditec AG, Jena, Germany) platform equipped with the ZEN 2012 software, lasers (488 or 561 nm), and 405 nm diode lamp. Images were optimised for colour, brightness and contrast for best clarity. Multiple-channel images were overlaid using ZEN light software. Immunohistochemistry studies were performed in the Laboratory of Advanced Microscopy Techniques MMRC PAS.

### Western Immunoblotting

Analysis of immunoreactivity of proteins was performed as described previously (Wilkaniec et al., 2018). Tissue samples were homogenised, mixed with Laemmli buffer and denatured at 95°C for 5 min. After SDS-PAGE, proteins were transferred to a nitrocellulose membrane in standard conditions and used for immunochemical analysis, followed by chemiluminescent detection. The membranes were stripped (25 mM Glycine-HCl, 1% (w/v) SDS, pH 2; 30 min at room temperature) and re-probed. As the first, phosphorylated protein was immunodetected, then the total level, and finally, GAPDH or vinculin. Glyceraldehyde 3-phosphate dehydrogenase (GAPDH) or vinculin level was analysed as a loading control. In all experiments, densitometric analysis of immunoblots was performed using normalisation to immunoreactivity of GAPDH or vinculin. Densitometric analysis and size-marker based verification was performed with TotalLab4 software (NonLinear Dynamics Ltd., Newcastle upon Tyne, UK).

### Measurement of Cytokine Levels in Brain Tissue Extract

Hippocampi were homogenised in ice-cold buffer (20 mM Tris HCl, 0.15 M NaCl, 2 mM EDTA, 1 mM EGTA, and Protease Inhibitor Cocktail) and centrifuged (4,500 × g, 5 min, 4°C). The resulting supernatant was used to determine cytokines level of EPO, G-CSF, GM-CSF, GRO/KC, IFN-γ, IL-1α, IL-1β, IL-2, IL-4, IL-5, IL-6, IL-7, IL-10, IL-12p40, IL-12p70, IL-13, IL-17A, IL-18, MCP-1, M-CSF, MIP-1α, MIP-2, MIP-3α, RANTES, TNF-α, VEGF, by using Bio-Plex Pro™ Rat Cytokine 23-Plex Assay on the Luminex Bio-Plex 200 system (Bio-Rad Laboratories) according to the manufacturer's instructions. Data were calculated by generating a calibration curve obtained using recombinant cytokines. Cytokines that were not detected were assigned a value of zero in al analyses. Data were normalised to protein level.

### Determination of Glutathione Levels

Total (GSSG+GSH) and oxidised glutathione (GSSG) levels were measured using an enzymatic assay kit (Cayman Chemical, Ann Arbour, USA) as described previously (Dominiak et al., 2017). Tissues were homogenised in ice-cold buffer (50 mM MES, pH 6–7; 1 mM EDTA) and centrifuged (10,000 × g, 15 min, 4°C). The resulting supernatant was used to determine protein content, and then it was deproteinated. GSSG concentration was determined by the derivatization technique according to the manufacturer's instructions. The reaction was initiated by adding a freshly prepared assay cocktail, and the change in absorbance was detected on a microplate reader Multiscan GO (Thermo Scientific) at 405 nm after 25 min. The results were normalised to protein level.

### Measurement of the Reactive Oxygen Species (ROS) Level

Measurement of the ROS level was carried out using fluorescent probe 2′,7′-dichlorodihydrofluorescein diacetate (DCFH-DA), as described previously (Dominiak et al., 2017). DCFH-DA is deacetylated by cellular esterases to 2′,7′-dichlorodihydrofluorescein (DCFH) and then may be oxidised to a highly fluorescent compound, 2′,7′-dichlorofluorescein (DCF). Homogenate (1% in PBS) of hippocampal tissue was incubated in the dark in the presence of 10 μM DCFH-DA at 37°C for 45 min. DCF fluorescence was measured using a microplate reader TECAN Infinite M1000PRO at 488 nm excitation and 525 nm emission wavelengths. Each sample was analysed in triplicate. To confirm that deacetylation of probe was not a limiting factor of reaction, each sample was incubated additionally in the presence of 10 μM FeCl_2_ (positive control). The results of fluorescence measurements are presented as arbitrary units (AUs).

### Mitochondria Isolation and Determination of the Mitochondrial Membrane Potential (ΔΨm)

Isolation of intact mitochondria from hippocampi of adolescent rats was performed using the Mitochondrial Isolation kit (MITOISO1; Sigma-Aldrich). After tissue homogenization in extraction buffer A (10 mM HEPES, pH 7.5; 200 mM mannitol; 70 mM sucrose; 1 mM EGTA), centrifugation at low (600 × g for 5 min) and high speed (11,000 × g for 10 min) was performed. Isolated mitochondria were resuspended in 40 μl storage buffer (10 mM HEPES, pH 7.5, containing 250 mM sucrose, 1 mM ATP, 80 μM ADP, 5 mM sodium succinate, 2 mM K_2_HPO_4_, 1 mM DTT) and used directly for JC-1 staining.

Detection of mitochondrial membrane potential (ΔΨm) (inner membrane integrity) was achieved by JC-1 uptake according to the manufacturer's directions. JC-1 (5′,6,6′-tetrachloro-1,1′,3,3′-tetraethylbenzimidazolylcarbocyanine iodide) exhibits potential-dependent accumulation in mitochondria, indicated by a fluorescence emission shift from green (527 nm) to red-orange (590 nm). The fluorescence of the mitochondria sample was read in a spectrofluorometer TECAN Infinite M1000PRO with settings as follows: excitation wavelength = 490 nm; slit = 5 nm and emission wavelength = 590 nm; slit = 7.2 nm. The mitochondrial membrane potential was expressed as fluorescence produced in the mitochondria suspension per milligramme of mitochondrial protein (FLU/mgP).

### Complex I Activity Assay

Mitochondrial OXPHOS complex I activity was determined by immunocapture ELISA according to the manufacturer's protocol (complex I enzyme activity microplate assay kit, Abcam). Briefly, 50 μg of protein from tissue extracts was incubated in the wells of a microplate, which was pre-coated with complex I capture antibody, for 3 h at RT. Then, activity of the immunocaptured complex I enzyme was determined by measuring the oxidation of NADH to NAD^+^ and simultaneous reduction of a dye which leads to increased absorbance at 450 nm. Each sample was measured on a microplate reader Multiscan GO (Thermo Scientific) in triplicate, and the activity was expressed as change in absorbance per minute per amount of sample loaded into the well.

### Complex IV Activity Assay

Mitochondrial OXPHOS complex IV activity was measured as an activity of the cytochrome c oxidase enzyme (EC 1.9.3.1) in a hippocampal tissue using a Complex IV Rodent Enzyme Activity Microplate Assay Kit (Abcam, USA) according to the manufacturer's instructions. The enzyme was immunocaptured within the wells of the microplate, and its activity was determined colourimetrically by following the oxidation of reduced cytochrome c, as the absorbance decreased at 550 nm. Each sample was measured on a microplate reader Multiscan GO (Thermo Scientific) in triplicate, and the activity was determined by calculating the slope between two points within the linear region.

### Analysis of the mRNA Level

Analysis of mRNA level was performed as described previously (Wilkaniec et al., 2018). Total RNA was isolated by using TRI reagent according to the manufacturer's protocol, and digestion of DNA contamination was performed by using DNase I according to the manufacturer's protocol (Sigma Aldrich, St. Louis, MO, USA). RNA quantity and quality were controlled by spectrophotometric analysis and gel electrophoresis. A reverse transcription was performed by using the High Capacity cDNA Reverse Transcription Kit according to the manufacturer's protocol (Thermo Fisher Scientific). Quantitative PCR was performed on an ABI PRISM 7500 apparatus using the commercially available TaqMan Gene Expression Assays: *Vamp1* (Rn00565308_m1), *Vamp2* (Rn00360268_g1), *Syp* (Rn01528256_m1), *Syn1* (Rn00569468_m1), *Syt1* (Rn 00436862_m1), *mt-Nd1* (Rn03296764_s1), *Sdha* (Rn00590475_m1), *mt-Cyb* (Rn03296746_s1), *mt-Co1* (Rn03296721_s1), *Sod1* (Rn00566938_m1), *Sod2* (Rn00566942_g1), *Ifng* (Rn00594078_m1)*, Il6* (Rn01410330_m1), *Il1b* (Rn00580432_m1), *Tnf* (Rn99999017_m1), *Chi3l1* (Rn01490608_m1), *Mrc1* (Rn01487342_m1), *Il10* (Rn99999012_m1), *Cd86* (Rn00571654_m1), *Fcgr1a* (Rn01762682_m1), *Tgfb1* (Rn00572010_m1), *Sphk1* (Rn00591307_m1), and *Actb* (Rn00667869_m1) as the reference gene, according to the manufacturer's instructions (Thermo Fisher Scientific). The relative levels of mRNA were calculated by using the ΔΔCt method.

### Determination of Protein Level

Concentration of proteins in samples was determined using the Pierce™ BCA Protein Assay Kit (Thermo Fisher Scientific) according to the manufacturer's instructions, with BSA as a standard. Each measurement was performed on a microplate reader Multiscan GO (Thermo Scientific) in duplicate at 562 nm absorbance.

### Statistical Analysis

The results were expressed as mean values ± S.E.M. In all analyses, each data-point is from separate animal. The normality and equality of group variances were tested by a Shapiro–Wilk test. Differences between means were analysed using unpaired Student's *t*-test. Level of statistical significance was set at *p* < 0.05. The statistical analyses were performed using Graph Pad Prism version 6.0 (Graph Pad Software, San Diego, CA).

## Results

### MIA Stimulates an Increase of Inflammatory Markers in Foetuses and Placenta

Our model of MIA in rats involved the use of LPS applied to pregnant dams at GD9.5. An LPS dose of 100 μg/kg b.w. was shown previously to induce a short-term rise of oxidative stress and pro-inflammatory cytokines in the brain and blood of adult female rats (Dominiak et al., 2017). LPS evoked in foetuses and placenta an increase in the expression of some inflammation-related genes as well as an increase in the level of Iba1-a marker of microglia (Supplementary Figure 1). Transient sickness behaviour, including changes in water intake and body temperature, was also observed in LPS-injected dams (Cieślik et al., 2020).

### MIA Induces Ultrastructural Changes in the Hippocampus of Rat Offspring

**Figure 1:**
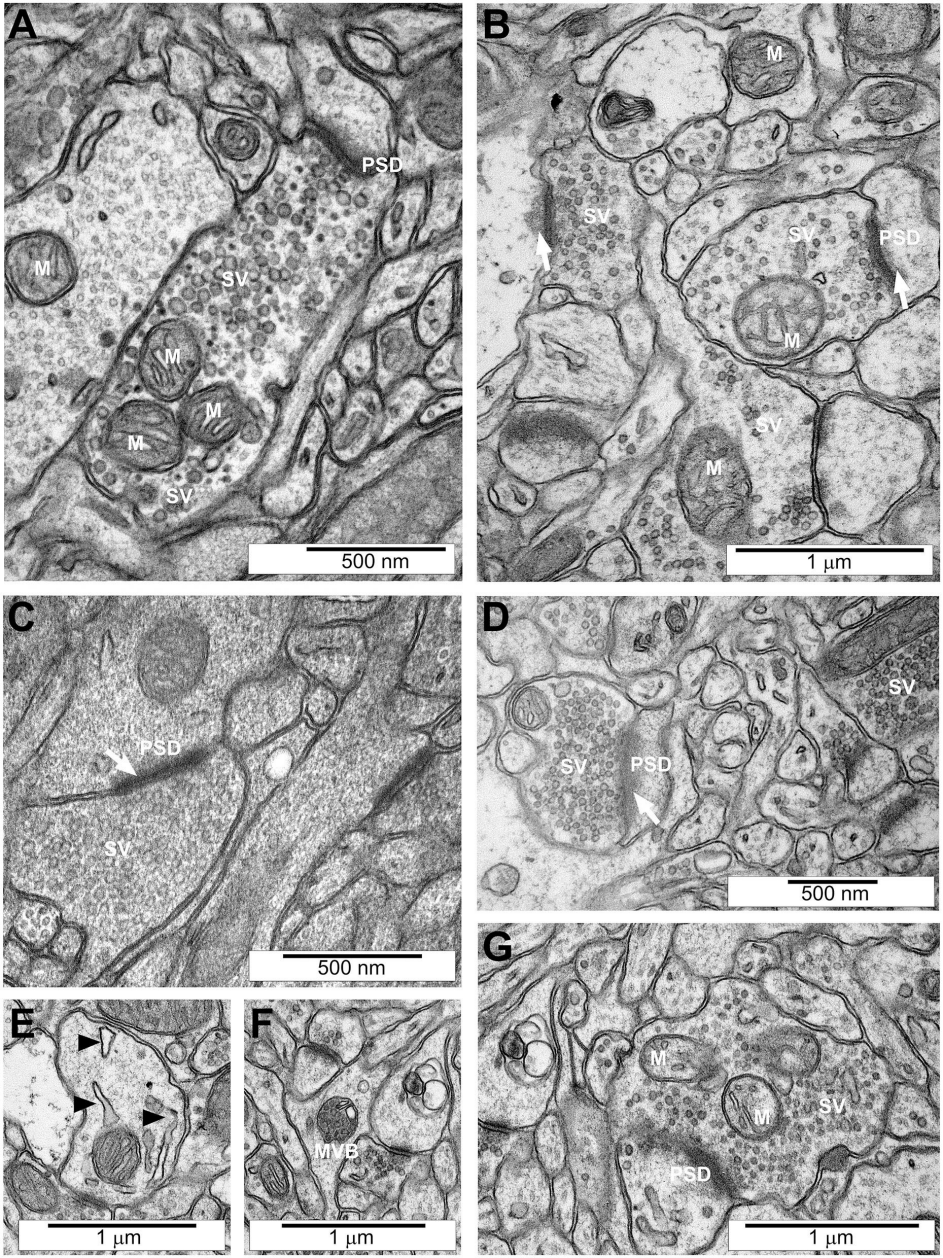
Ultrastructure of neuronal cells in the hippocampi of control and MIA-affected offspring. LPS (100 μg/kg b.w.) was injected intraperitoneally at gestation day 9.5 to female rats. Ultrastructure of male offspring's hippocampi was analysed at day 52. **(A)** Control group. Ultrastructurally unchanged neuronal cells, unaltered structure of neuropil, well-defined structure of synapses with accurate post-synaptic density (PSD) and correct distribution of synaptic vesicles (SVs), well-preserved mitochondria (M). **(B–G)** MIA-exposed group. Neural cells and neuropil with features of swelling are observed. Reduced packing density of SVs in presynaptic area **(B)**, blurred and thickened structure of synaptic cleft (arrows) **(B–D)**, disturbed synaptic membrane with free SVs **(G)**, swollen mitochondria **(B)**, swollen endoplasmic reticulum (arrowheads on **E**) and multi-vascular bodies (MVBs) **(F)**. Representative pictures are presented.

It was observed that LPS-evoked MIA induced significant alterations in the brains (cornu ammonis subregions CA2 and CA3) of adolescent offspring rats. The TEM analysis of hippocampi of control rats showed unchanged ultrastructure of synapses, synaptic vesicles (SVs), and mitochondria (Figure 1A). Visible synapses had the proper distribution of SVs in the cytoplasm. The synaptic cleft was narrow and the postsynaptic density (PSD) was prominent and clearly stained. The nerve endings did not reveal the features of the swelling. However, the images of the brain tissue of rats prenatally exposed to LPS clearly demonstrated ultrastructural changes in synapses (Figures 1B–G). Diminished packing density of SVs in the presynaptic area (Figure 1B) as well as blurred and thickened structure of the synaptic cleft (arrows on Figures 1B–D) were observed in MIA animals. Moreover, disturbed synaptic membrane with free SVs (Figure 1G), swollen mitochondria (Figure 1B), swollen endoplasmic reticulum (arrowheads on Figure 1E) and multivesicular bodies (MVBs) were present in the examined structure (Figure 1F). Pathological changes were limited to nerve endings.

**Figure 2:**
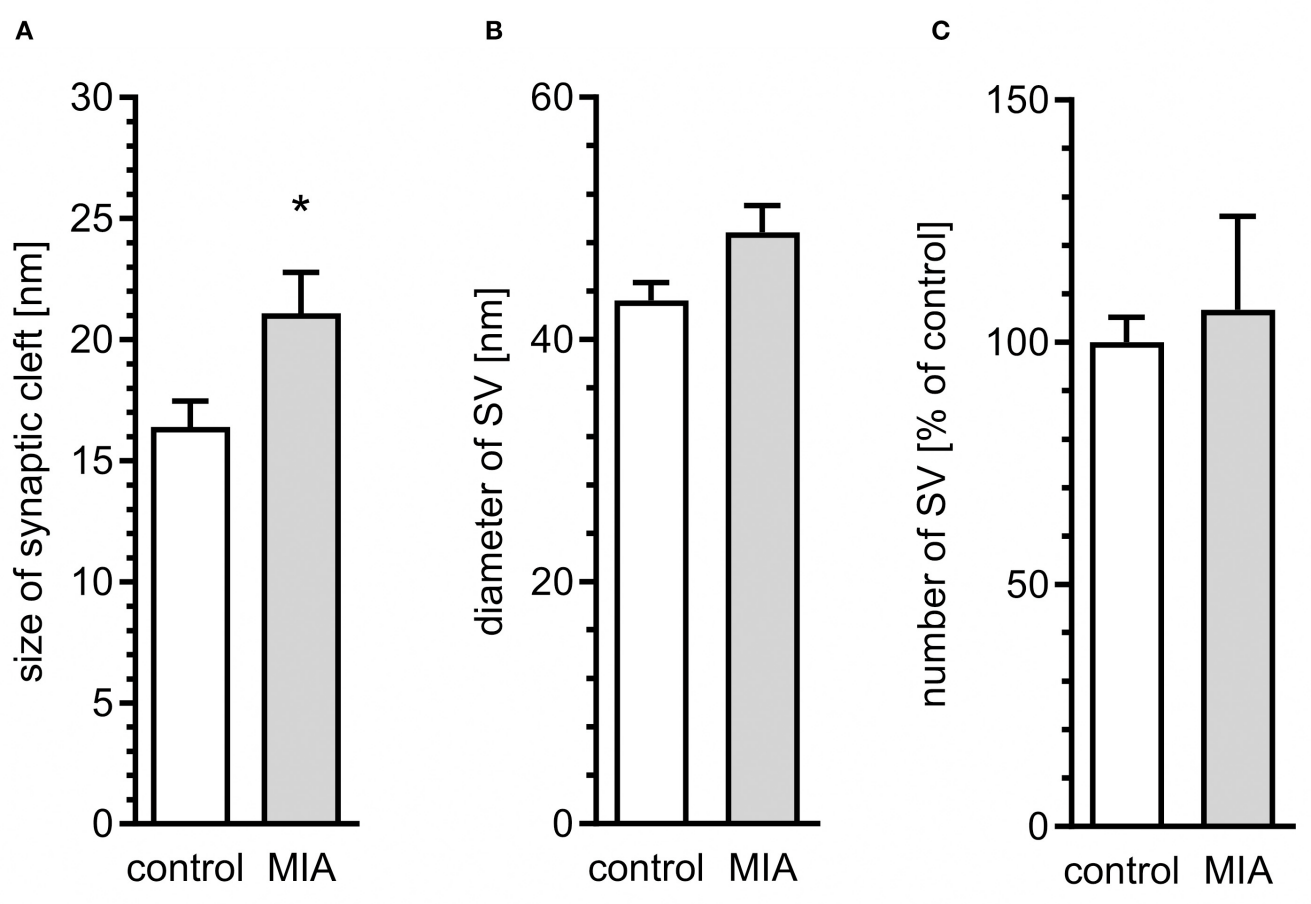
Quantitative analysis of ultrastructural alterations of the synapse in adolescent male rats exposed prenatally to maternal immune activation. LPS (100 μg/kg b.w.) was injected intraperitoneally at gestation day 9.5 to female rats. Ultrastructure of male offspring's hippocampi was analyzed at day 52. Quantitative analysis of **(A)** size of synaptic cleft (*n* = 7 in control and 3 in MIA group), **(B)** diameter (*n* = 3 in both groups), and **(C)** number of synaptic vesicles (*n* = 5 and 3) was performed. *p < 0.05, compared with the control group.

Quantitative analysis of microphotographs confirmed ultrastructural changes in synapses. The average size of the synaptic cleft was increased in MIA-affected offspring, as compared to control animals (Figure 2A). However, diameter of SVs and the number of SVs were not changed (Figures 2B,C). These data indicate that synaptic organisation is changed in MIA-affected offspring, suggesting that synaptic function may be affected. Our previous studies demonstrated that stress evoked by environmental factors such as LPS-evoked systemic inflammation or perinatal exposure to lead (Pb) or LPS induces ultrastructural and molecular alterations in synapses of rat offspring (Czapski et al., 2007, 2010; Ga̧ssowska et al., 2016b; Cieślik et al., 2020). It was also shown that neuroinflammatory processes in young septic rats may decrease the level of synaptophysin and change the structure of cortical synapses (Han et al., 2017).

### MIA Alters Expression, Level and Phosphorylation of Pre- and Post-Synaptic Proteins in the Hippocampus of Rat Offspring

**Figure 3:**
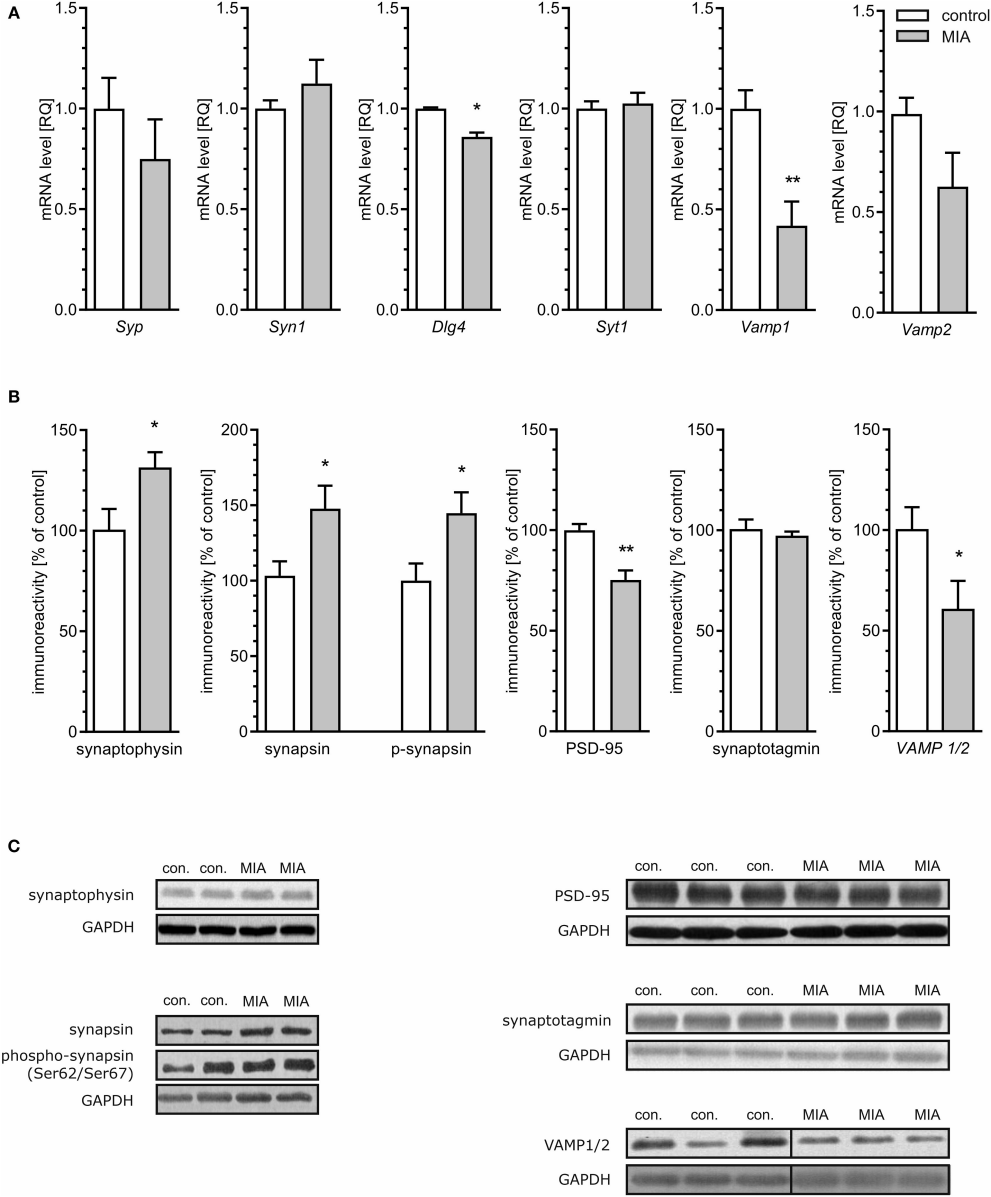
Maternal immune activation alters the expression, level, and phosphorylation of synaptic proteins in hippocampi of adolescent male rats. LPS (100 μg/kg b.w.) was injected intraperitoneally at gestation day 9.5 to female rats. Offspring male rats at day 52 were decapitated, and the hippocampi were collected. **(A)** The level of mRNA for synaptophysin (*Syp*; *n* = 5), synapsin (*Syn1*; *n* = 5), PSD-95 (*Dlg4*; *n* = 4), synaptotagmin (*Syt1*; *n* = 5), synaptobrevins/VAMPs (*Vamp1*; *n* = 4; *Vamp2*; *n* = 4 and 5). The level of mRNA was measured by real-time PCR and calculated by the ΔΔCt method with *Actb* as a reference gene. **(B)** The level of immunoreactivity of synaptophysin (*n* = 4), synapsin (*n* = 3 and 4) and phospho-synapsin (Ser62/Ser67) (*n* = 5 and 4), PSD-95 (*n* = 5), synaptotagmin (*n* = 4), and synaptobrevin/VAMP1/2 (*n* = 8 and 7). Densitometric analysis was performed using normalisation to immunoreactivity of GAPDH. **(C)** Representative immunoblots including GAPDH as a loading control. Each sample presented on representative pictures is from separate animal. Immunoblots for synaptobrevin/VAMP1/2 show non-adjacent bands from the same blot without any additional manipulation. Vertical black lines show position of deleted area, which contained unrelated experimental group. *p < 0.05, **p < 0.01, compared with the control group.

To evaluate if morphological alterations of synapses in hippocampi of MIA-affected offspring may be related to changes of synaptic proteins, quantitative analysis of the mRNA level, and semi-quantitative analysis of the level of selected pre- and post-synaptic proteins was performed. As shown in Figure 3A, the hippocampal expression of some genes coding synaptic proteins was changed in MIA offspring rats. The level of mRNA for synaptophysin (*Syp*), synapsin (*Syn1*), synaptotagmin (*Syt1*), and VAMP2 (*Vamp2*) was not changed; however, the mRNA level for PSD-95 (*Dlg4*) and VAMP1 (*Vamp1*) was reduced. Additionally, protein level and phosphorylation were altered by MIA (Figures 3B,C). The level of both synaptophysin and synapsin, as well as phosphorylated form of synapsin (Ser62/67), was significantly increased. Analysis of the postsynaptic protein PSD-95 revealed that in MIA- affected offspring, the level of this protein was reduced. Likewise, the anti-VAMP1/2 immunoreactivity was decreased. The protein levels of synaptotagmin, syntaxin-1, and SNAP-25 (Supplementary Figure 2A) were not changed.

### MIA Alters Level and Phosphorylation of MAPT and MAPT-Related Kinases in the Hippocampus of Rat Offspring

**Figure 4:**
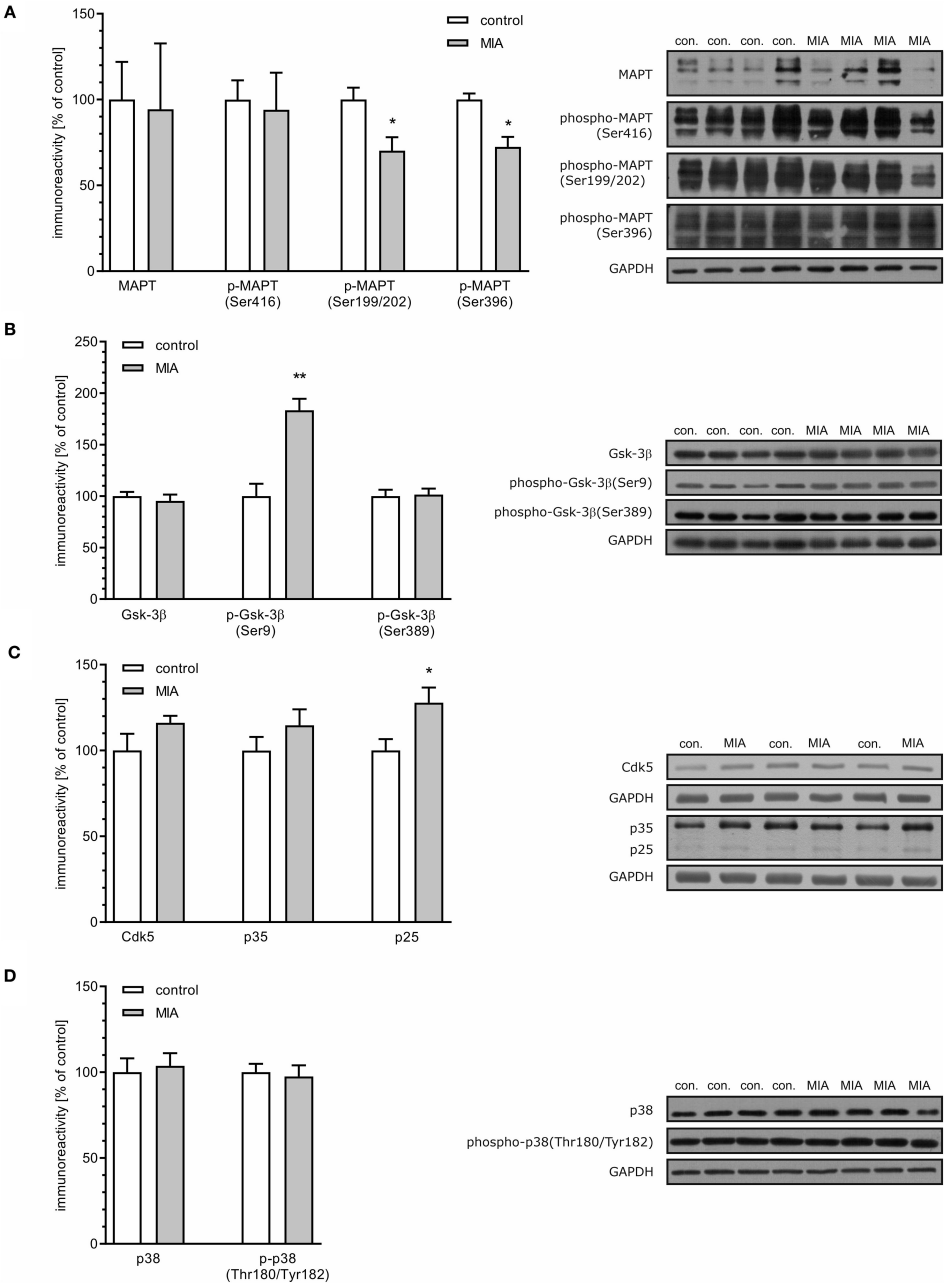
Maternal immune activation induces alteration of phosphorylation level of MAPT, Gsk-3β, Cdk5, p35/p25, and p38 in hippocampi of adolescent male rats. LPS (100 μg/kg b.w.) was injected intraperitoneally at gestation day 9.5 to female rats. Offspring male rats at day 52 were decapitated, and the hippocampi were collected. **(A)** Densitometric analysis of total MAPT (*n* = 4), phospho-MAPT (Ser416) (*n* = 5), phospho-MAPT (Ser199/202) (*n* = 4 and 5), phospho-MAPT (Ser396) (*n* = 4). **(B)** Densitometric analysis of Gsk-3β (*n* = 4 and 5), phospho-Gsk-3β (Ser9) (*n* = 4), and phospho-Gsk-3β (Ser389) (*n* = 5). **(C)** Densitometric analysis of Cdk5 (*n* = 6) and p35/p25 (*n* = 6). **(D)** Densitometric analysis of p38 (*n* = 9) and phospho-p38 (Thr180/Tyr182) (*n* = 9). Data were normalised to GAPDH immunoreactivity. Representative immunoblots are presented, including GAPDH as a loading control. Each sample presented on representative pictures is from separate animal. *p < 0.05, **p < 0.01, compared with the control group.

Recent studies demonstrated that presynaptic dysfunction may be evoked by deregulation of MAPT (Moreno et al., 2016; Zhou et al., 2017). As demonstrated in Figure 4A, the total level of MAPT was not changed in the hippocampi of MIA-affected offspring. Similarly, phosphorylation at Ser416 was not altered. However, phosphorylation of MAPT at Ser199/202 and Ser396 was significantly reduced.

Both Ser199/202 and Ser396 on MAPT are phosphorylated by glycogen synthase kinase 3β (Gsk-3β) and cyclin-dependent kinase 5 (Cdk5); therefore, reduced phosphorylation of MAPT may be an indication of reduced activity of these kinases. However, reduced phosphorylation may be also a consequence of enhanced dephosphorylation evoked by oxidative stress and catalysed by protein phosphatase 1 (PP1) (Zambrano et al., 2004; Szatmari et al., 2005; Hernandez et al., 2010). To cheque these possibilities, we first analysed the level and phosphorylation of Gsk-3β on Ser9 and Ser 389, which are molecular marks of deactivation of Gsk-3β (Thornton et al., 2008). In accordance with changes in phosphorylation of MAPT, phosphorylation of Ser9 was significantly increased, which indicates that the activity of this kinase is suppressed, even if total level of Gsk-3β was not changed (Figure 4B). Phosphorylation of Gsk-3β at Ser389 was not changed (Figure 4B).

Cdk5 is a serine-threonine kinase that is directly responsible for phosphorylation of MAPT and indirectly for phosphorylation of Gsk-3β (Czapski et al., 2016). To achieve catalytic activity, Cdk5 must bind its regulatory protein p35. In stress conditions, p35 may be cleaved by calcium-dependent calpains, leading to formation of p25, capable of overactivation, and mislocalization of Cdk5. The levels of Cdk5 and p35 were unaltered in MIA-affected offspring; however, the level of p25 was increased (Figure 4C), suggesting that activity of Cdk5 increased. The enhanced activity of Cdk5 suggests that it cannot be responsible for hypophosphorylation of MAPT. However, Cdk5 can also indirectly evoke phosphorylation of Gsk-3β on Ser9, reducing Gsk-3β's activity as a tau kinase. Gsk-3β kinase activity is also negatively regulated by the p38 MAPK. Therefore, we analysed phosphorylation of p38 at the regulatory activation site (Thr180/Tyr182) (Raingeaud et al., 1995). In accordance with data showing unchanged immunoreactivity of phospho-Gsk-3β (Ser389), phosphorylation of p38 at Thr180/Tyr182 was not increased, indicating that p38 cannot be responsible for phosphorylation and inactivation of Gsk-3β (Figure 4D). Overall, these data suggest that hypophosphorylation of MAPT might be linked to Cdk5-dependent inhibition of Gsk-3β.

### MIA Induces Oxidative Stress in the Hippocampus of Rat Offspring

**Figure 5:**
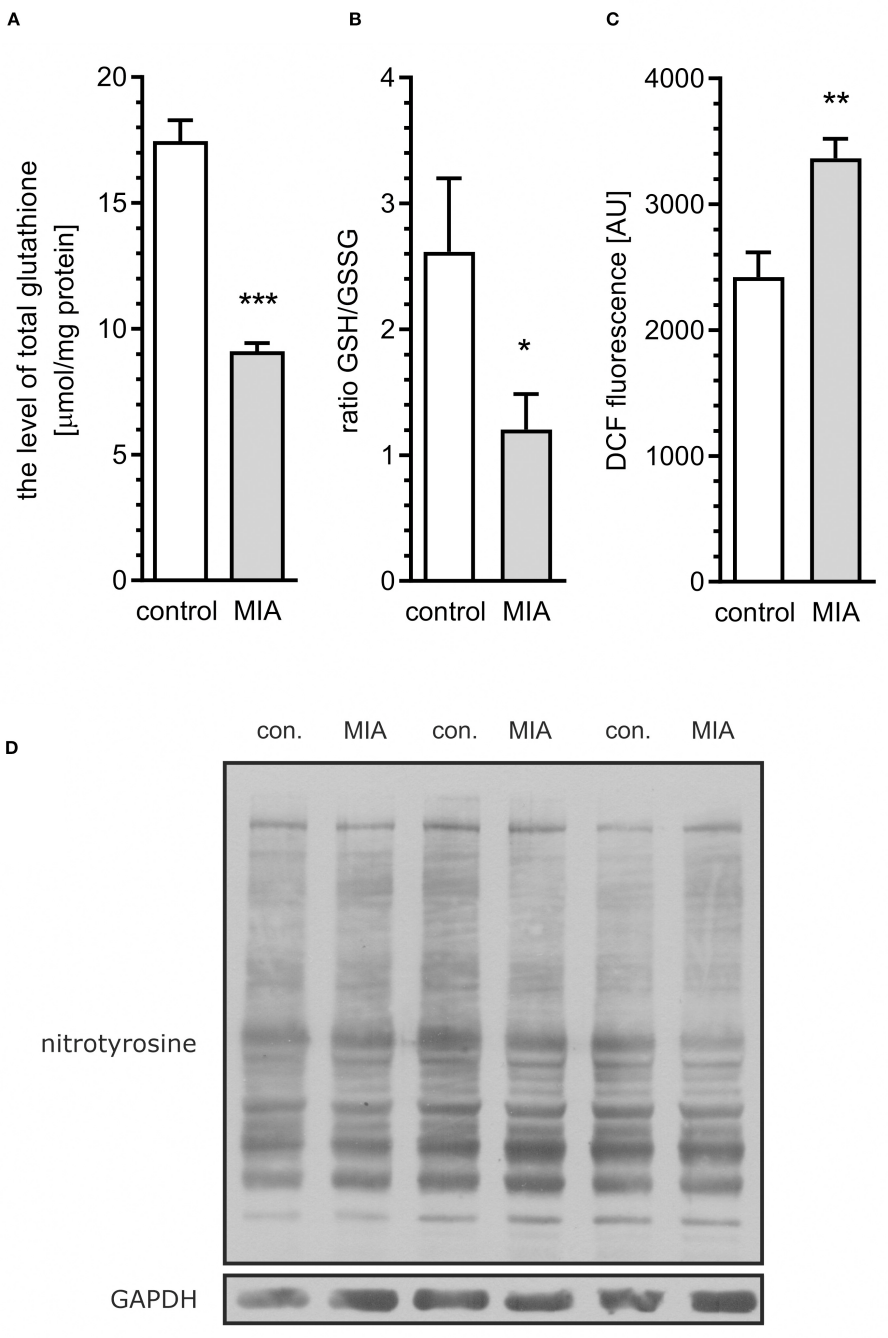
Maternal immune activation evokes oxidative stress in hippocampi of adolescent male rats. LPS (100 μg/kg b.w.) was injected intraperitoneally at gestation day 9.5 to female rats. Offspring male rats at day 52 were decapitated, the hippocampi were collected and the level of total glutathione, oxidised glutathione (GSSG), and reduced glutathione (GSH) was measured. **(A)** Total glutathione (*n* = 7). **(B)** The oxidised/reduced glutathione ratio (*n* = 6). **(C)** ROS level in tissue homogenate (*n* = 8). **(D)** Representative immunoblots of nitrotyrosine, including GAPDH as a loading control. Each sample presented on representative pictures is from separate animal. Densitometric analysis was performed using normalisation to immunoreactivity of GAPDH. *p < 0.05, **p < 0.01, ***p < 0.001, compared with the control group.

Because the previous study demonstrated that Cdk5-dependent, I-2/PP1-mediated dephosphorylation of MAPT in neuronal cells may by induced by oxidative stress (Zambrano et al., 2004), our next goal was to analyse whether oxidative stress is present in MIA-affected offspring. As shown in Figure 5A, total level of glutathione, the major antioxidant in the brain, was lowered in MIA-affected offspring. Moreover, the reduced/oxidised glutathione ratio was decreased (Figure 5B), indicating enhanced generation of ROS. For additional confirmation of oxidative stress, analysis of DCF fluorescence in hippocampal tissue homogenate was performed. This fluorogenic probe is oxidised by a wide range of ROS; therefore, it is used as a general measure of ROS formation and oxidative stress. As shown in Figure 5C, fluorescence of oxidised DCF in homogenate of hippocampal tissue from MIA offspring is about 40% higher than from control rats. The formation of free radicals and other reactive species is a significant event in numerous pathological conditions of CNS. In brains of MIA-affected offspring, the most probable sources of ROS are impaired mitochondria or cytotoxic mechanisms related to activated immune system (Sheng et al., 2013; Yui et al., 2015; Bjorklund et al., 2016). Increased accumulation of p25 (Figure 4C) proves calpain activation and Ca^2+^ dysregulation, which could suggest activation of microglia (Hoffmann et al., 2003). The main ROS produced by activated “cytotoxic” microglia are superoxide radicals ${\text{O}}_{2}^{\cdot -}$ (generated mostly by NADPH oxidase) and nitric oxide NO^·^ (generated by inducible nitric oxide synthase). These two free radicals may form highly reactive oxidant–peroxynitrite (ONOO^−^), which is responsible for damaging invading bacteria but also may contribute to damage of own cells. The marker of peroxynitrite formation, and therefore the proof of activation of cytotoxic phenotype of microglia, is formation of nitrotyrosine. Our results demonstrate that the level of nitrotyrosine is not changed in the hippocampi of MIA-affected offspring (Figure 5D), suggesting that oxidative stress might be not evoked by cytotoxic mechanisms of activated microglia.

### MIA Does Not Activate Cytotoxic Microglia in the Hippocampus of Rat Offspring

**Figure 6:**
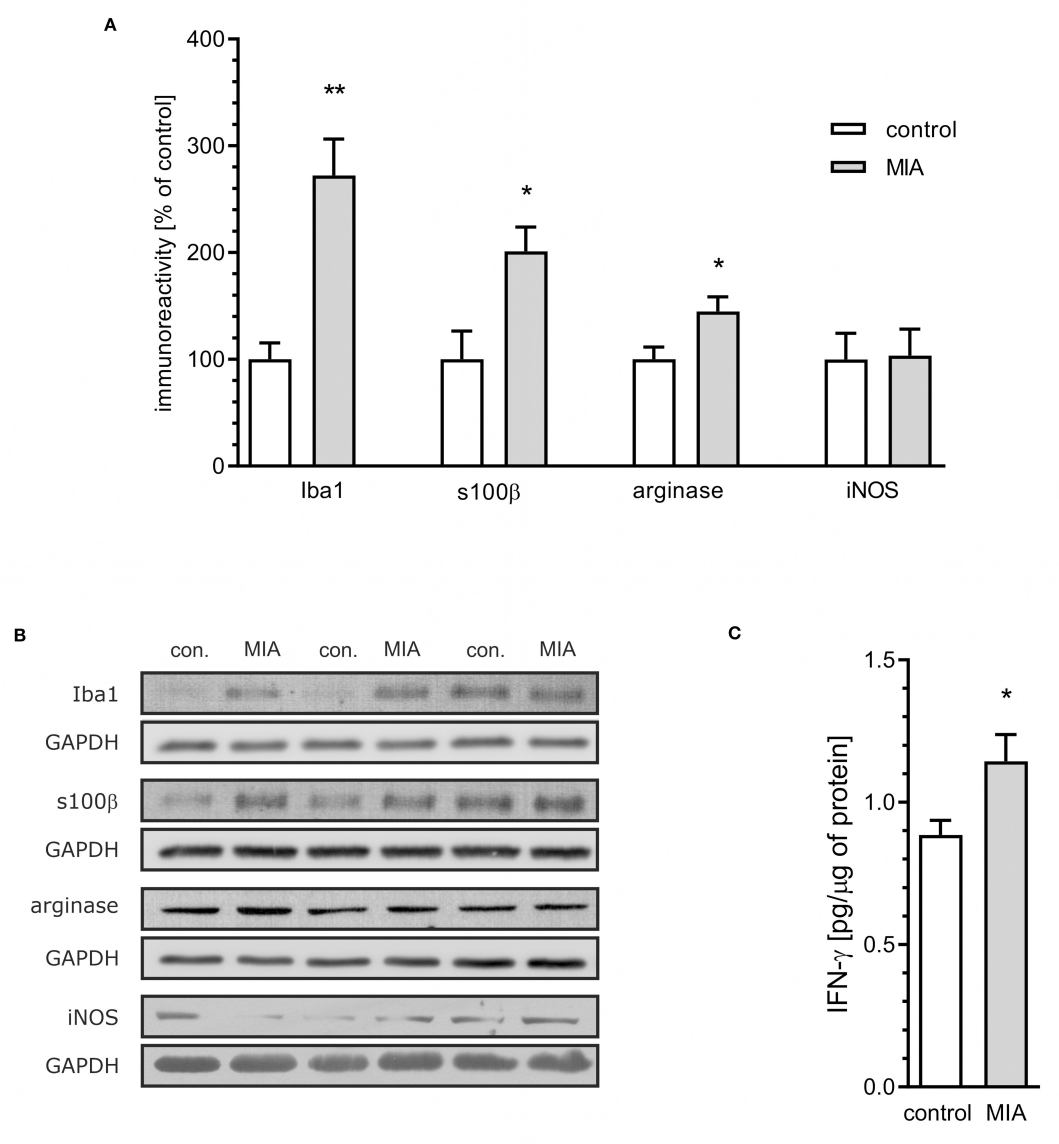
Maternal immune activation stimulates an increase of inflammatory markers in hippocampi of adolescent male rats. LPS (100 μg/kg b.w.) was injected intraperitoneally at gestation day 9.5 to female rats. Offspring male rats at day 52 were decapitated, and the hippocampi were collected. **(A)** Densitometric analysis of immunoreactivity of Iba1 (*n* = 3 and 4), s100β (*n* = 5 and 4), arginase (*n* = 5 and 4), and iNOS (*n* = 3 and 4). Data were normalised to GAPDH immunoreactivity. **(B)** Representative immunoblots of Iba1, s100β arginase and iNOS, including GAPDH as a loading control. Each sample presented on representative pictures is from separate animal. **(C)** The level of IFN-γ in hippocampal extract, as determined using the Bio-Plex Pro Rat Cytokine 23-Plex Assay on Luminex Bio-Plex 200 system (*n* = 7). *p < 0.05, **p < 0.01, compared with the control group.

**Figure 7:**
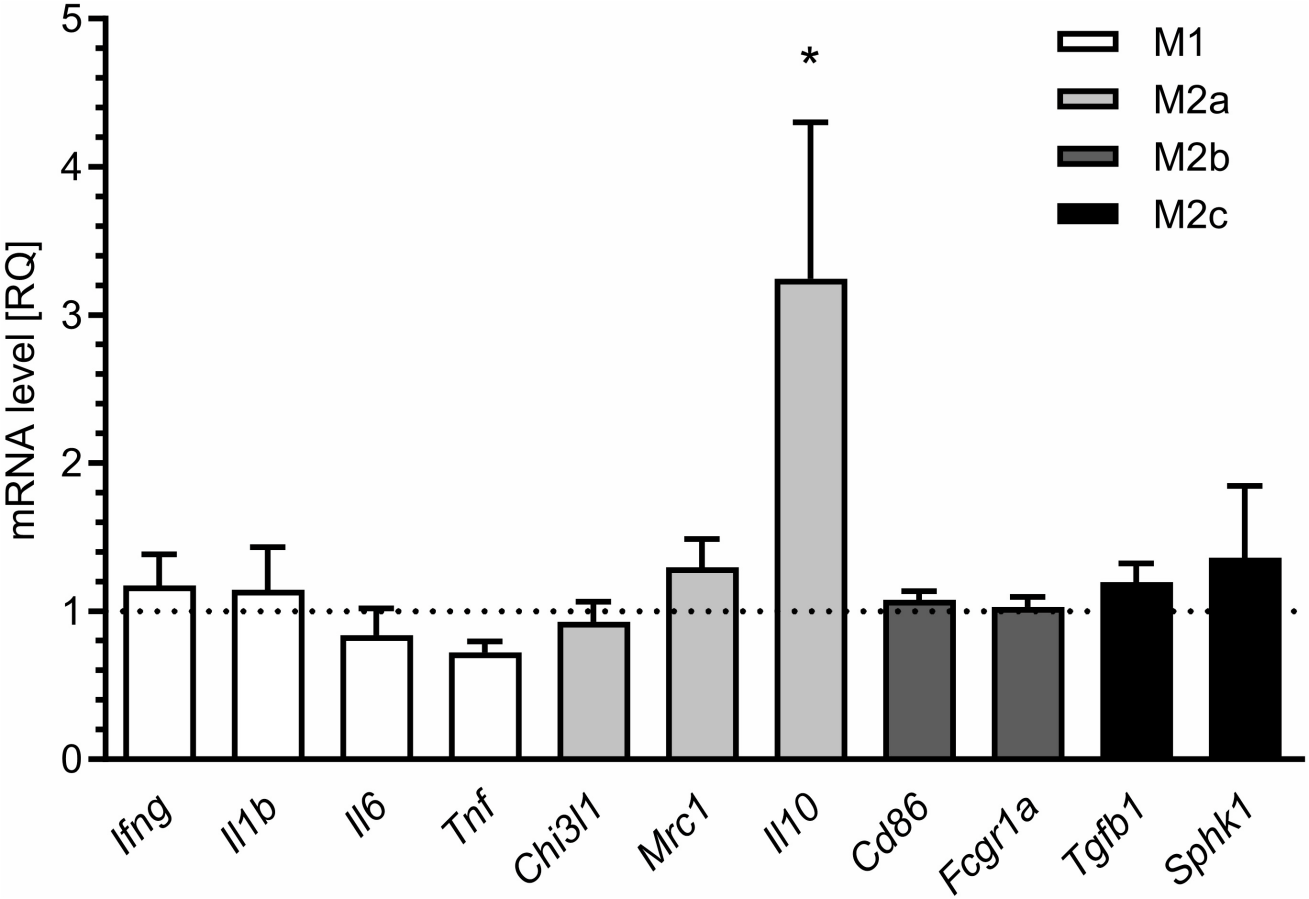
Maternal immune activation alters the expression of markers of microglial activation in hippocampi of adolescent male rats. LPS (100 μg/kg b.w.) was injected intraperitoneally at gestation day 9.5 to female rats. Offspring male rats at day 52 were decapitated, and the hippocampi were collected. The level of mRNA was measured by real-time PCR and calculated by the ΔΔCt method with *Actb* as a reference gene. *p < 0.05, compared with the control group (*n* = 5).

**Figure 8:**
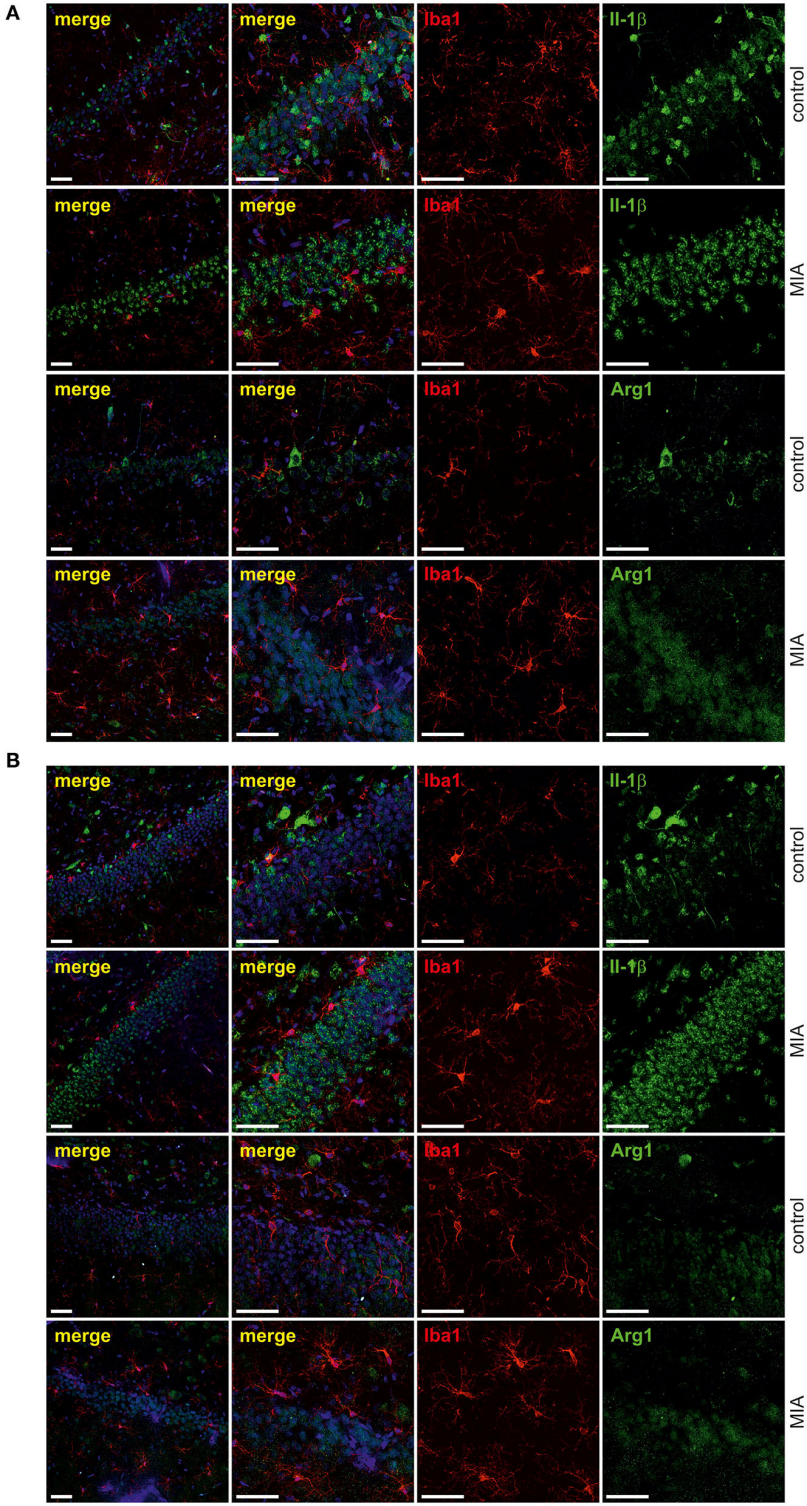
Effect of maternal immune activation on activation of microglia a in hippocampi of adolescent male rats. Immunohistochemical analysis illustrating microglia cells (Iba-1- red) in control and MIA-exposed groups in CA **(A)** and GD **(B)** regions of the hippocampus. No co-expression of activation markers, IL-1β (green) and arginase-1 (green), with Iba1-positive cells has been observed. The nuclei were counterstained with DAPI (blue). Scale bar = 50 μm.

**Figure 9:**
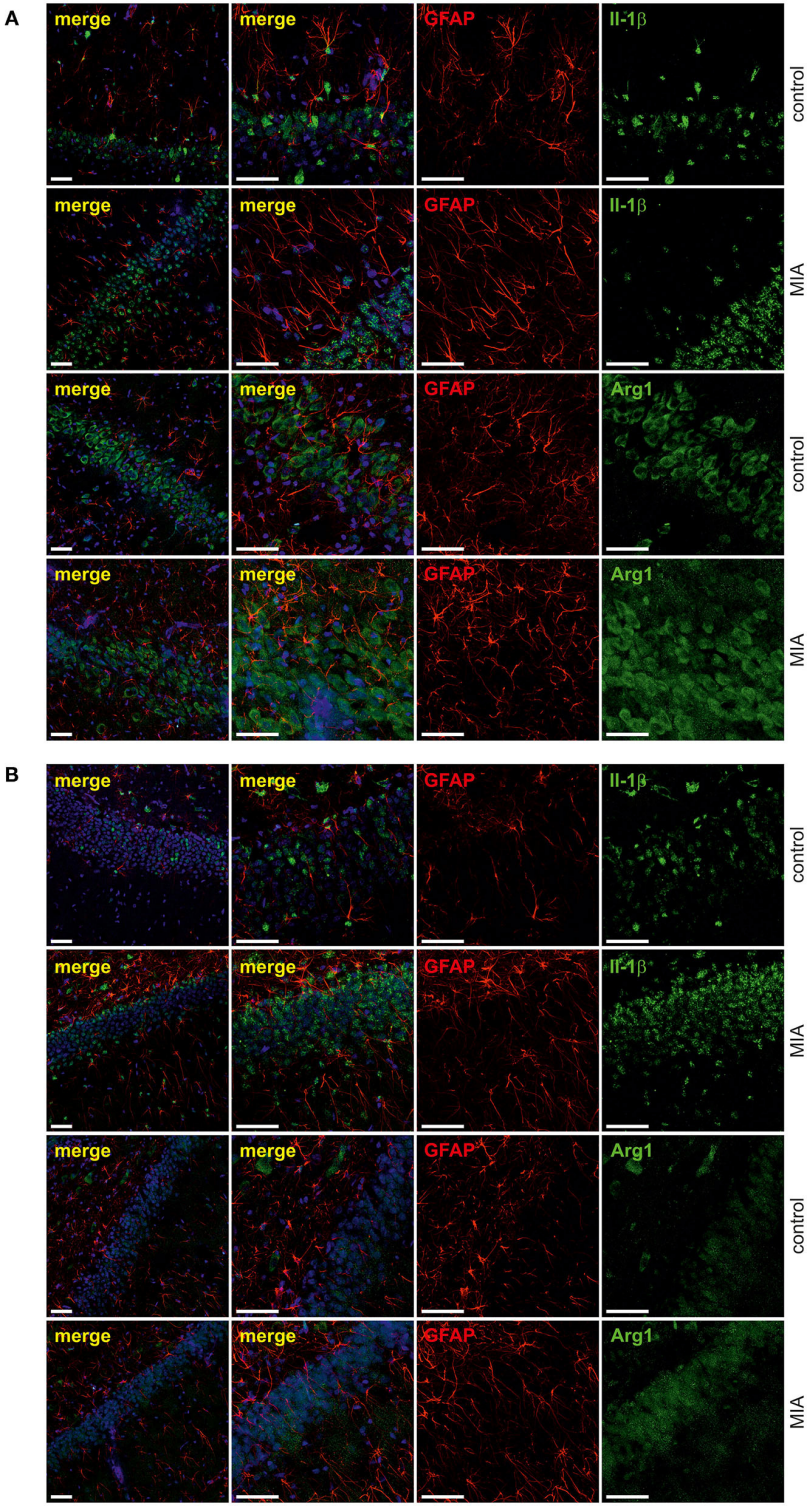
Effect of maternal immune activation on activation of astrocytes in hippocampi of adolescent male rats. Immunohistochemical analysis illustrating astrocytes (GFAP—red) in control and MIA-exposed groups in CA **(A)** and GD **(B)** regions of the hippocampus. No co-expression of activation markers, IL-1β (green) and arginase-1 (green), with GFAP-positive cells has been observed. The nuclei were counterstained with DAPI (blue). Scale bar = 50 μm.

For additional evaluation of the status of glial cells, we analysed two markers specific for microglia (Iba1) and astrocytes (s100β). We found that immunoreactivity of both Iba1 and s100β was elevated in the hippocampi of MIA-affected offspring (Figures 6A,B). To cheque if the enhanced level of Iba1 is the effect of activation or increased density of microglia, we analysed markers of microglial activation. In our experimental conditions, immunoreactivity of iNOS, the marker of M1, was not changed, but immunoreactivity of arginase, the marker of M2, was increased (Figures 6A,B). For deeper insight into the nature of neuroinflammatory processes in the hippocampus, the protein levels of pro- and anti-inflammatory cytokines in hippocampal tissue extract were evaluated by using immunoassay. Among tested cytokines (EPO, G-CSF, GM-CSF, GRO/KC, IFN-γ, IL-1α, IL-1β, IL-2, IL-4, IL-5, IL-6, IL-7, IL-10, IL-12p40, IL-12p70, IL-13, IL-17A, IL-18, MCP-1, M-CSF, MIP-1α, MIP-2, MIP-3α, RANTES, TNF-α, VEGF), only the level of IFN-γ was increased in MIA rats (Figure 6C, Supplementary Figure 3). In the next step, additional analysis of genetic markers of microglial activation was performed for detailed exploration of phenotype polarisation in the hippocampus (Sudduth et al., 2013). As shown in Figure 7, the only increase in mRNA level was observed for anti-inflammatory interleukin-10 (marker of phenotype M2a), whereas expression of genes for other phenotype markers was not enhanced. We also performed immunohistochemical analysis of proteins which are commonly recognised as markers of activation of microglia and astrocytes. As shown in Figures 8, 9, immunostaining of activation markers IL-1β and arginase-1 was not increased in microglia nor in astrocytes. These results exclude microglial iNOS as a notable source of ROS in the MIA-exposed rats.

### MIA Evokes Impairment of Mitochondrial Function in the Hippocampus of Rat Offspring

**Figure 10:**
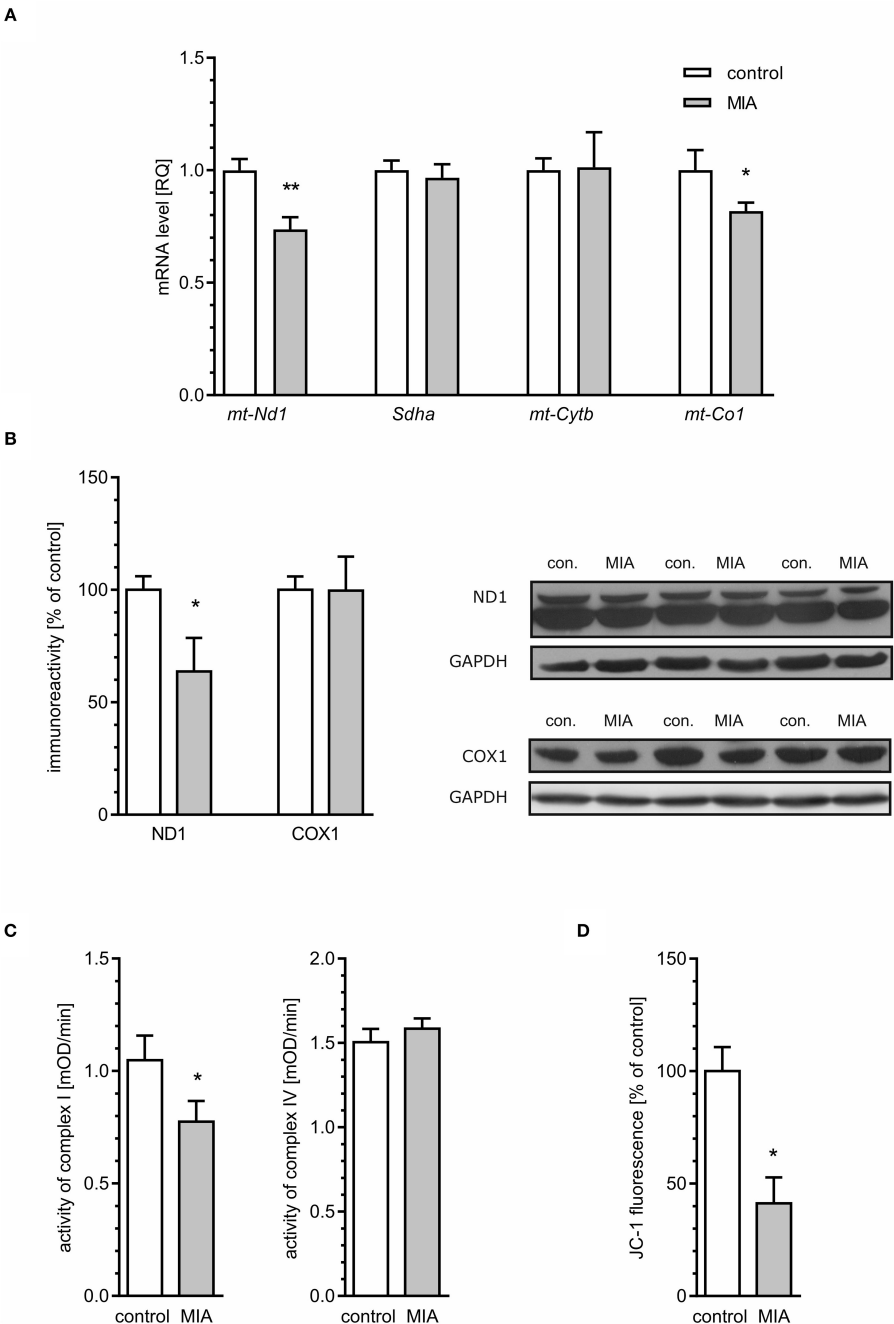
Maternal immune activation alters mitochondrial membrane potential and the expression and level of mitochondrial electron transport chain complexes in hippocampi of adolescent male rats. LPS (100 μg/kg b.w.) was injected intraperitoneally at gestation day 9.5 to female rats. Offspring male rats at day 52 were decapitated, and the hippocampi were collected. **(A)** The level of mRNA for *mt-Nd1* (*n* = 5 and 3), *Sdha* (*n* = 4), *mt-Cytb* (*n* = 5 and 4), and *mt-Co1* (*n* = 5) was measured by real-time PCR and calculated by the ΔΔCt method with *Actb* as a reference gene. **(B)** The level of proteins was analysed by the Western blot method. Densitometric analysis of ND1 (*n* = 7) and COX1 (*n* = 7 and 6) was performed using normalisation to immunoreactivity of GAPDH. Representative immunoblots, including GAPDH as a loading control, are presented. Each sample presented on representative pictures is from separate animal. **(C)** Activity of respiratory complexes I (*n* = 7 and 8) and IV (*n* = 8 and 7) was measured using the kinetic spectrophotometric method. **(D)** Mitochondrial membrane potential was determined by the fluorometric method using JC-1 (*n* = 3 and 8). *p < 0.05, **p < 0.01, compared with the control group.

The second likely source of ROS is mitochondrial dysfunction including electron transport chain (ETC) dysregulation (Hollis et al., 2017; Roberts, 2017; Joshi and Mochly-Rosen, 2018; Pei and Wallace, 2018). This hypothesis was supported and confirmed by analysis of mitochondrial ETC complexes. The levels of mRNA for *mt-Nd1* and *mt-Co1* genes (mitochondrially encoded subunits of complex I and IV, respectively) are reduced in MIA-affected offspring (Figure 10A). The expression of *Sdha* and *mt-Cytb* (subunits of complexes II and III, respectively) was not changed. To evaluate if these alterations impact protein level, immunoreactivity of ND1 and COX1 proteins in the hippocampus was analysed. As shown in Figure 10B, the immunoreactivity of ND1 is reduced in animals prenatally exposed to MIA, but we did not detect changes in the level of COX1. Then, the activity of respiratory complexes was measured using the kinetic spectrophotometric method. Based on the above data, we focused on complexes I and IV. As shown in Figure 10C, activity of complex I was reduced in the hippocampal tissue of MIA-affected offspring, but activity of complex IV was not altered. Finally, by using fluorescent probe JC-1, we analysed the mitochondrial membrane potential (MMP) in mitochondria isolated from hippocampal tissue (Figure 10D). Presented data clearly indicate a significant drop of MMP, suggesting severe impairment of mitochondrial function.

**Figure 11:**
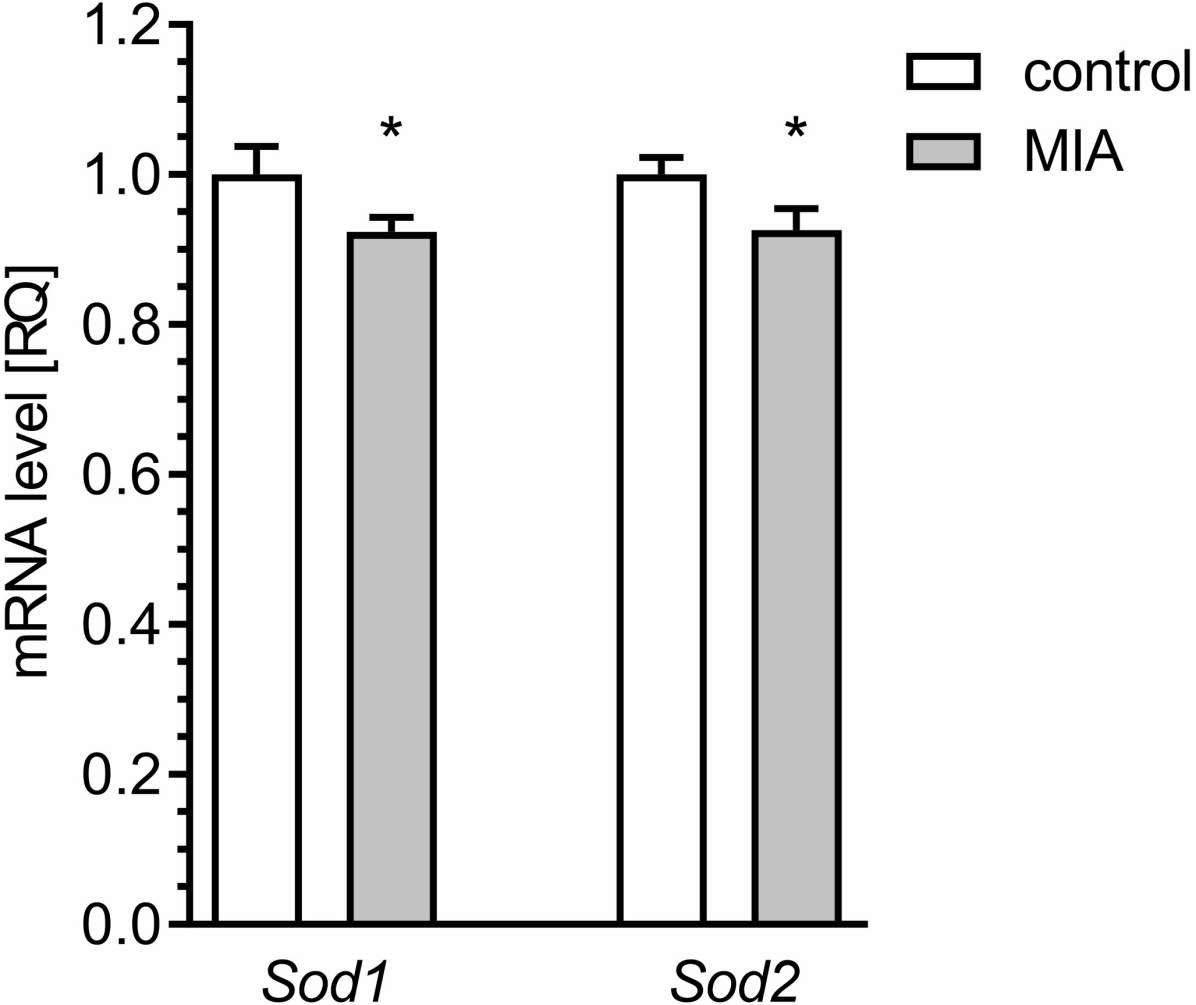
Maternal immune activation alters the expression of genes for superoxide dismutase 1 and 2 in hippocampus of adolescent male rats. LPS (100 μg/kg b.w.) was injected intraperitoneally at gestation day 9.5 to female rats. Offspring male rats at day 52 were decapitated, and the hippocampi were collected. The level of mRNA for *Sod1* (*n* = 4 and 5) and *Sod2* (*n* = 4 and 5) was measured by real-time PCR and calculated by the ΔΔCt method with *Actb* as a reference gene. *p < 0.05, compared with the control group.

An alternative basis for oxidative stress might be weakening of antioxidative defence. The major enzymatic mechanism of antioxidative defence in mitochondria is Mn-superoxide dismutase (Sod2), but the presence of some fraction of Cu, Zn-Sod (Sod1) in mitochondria was also reported. Our results demonstrated that the level of mRNA for both Sod1 and Sod2 was attenuated in the hippocampi of MIA-affected offspring (Figure 11).

## Discussion

In our recent study we demonstrated prolonged effects of MIA on neurobiological and behavioural parameters in offspring (Cieślik et al., 2020). Adolescent MIA offspring showed elevated blood cytokine levels, microglial activation, increased pro-inflammatory cytokines expression, and increased oxidative stress in the cerebral cortex. MIA-induced long-term changes in synaptic structure and pre-/post-synaptic proteins' levels. It was suggested that these changes could contribute to impairment of neuronal function and, in consequence, to behavioural abnormalities typical for ASD, i.e., alterations in social behaviour and communication.

In the current study, we focused on the hippocampus, which also was shown previously to be sensitive to inflammatory insults in both early pregnant and adult life, which may be a part of the pathogenesis of neurodevelopmental disorders including autism (Semmler et al., 2005; Makinodan et al., 2008; Czapski et al., 2010, 2013; Ito et al., 2010; Cavalier et al., 2019). Our study was aimed at evaluating the MIA-evoked molecular mechanism contributing to synaptic alterations in the hippocampi of offspring.

Our results demonstrated the changes in mitochondrial function in the hippocampi of MIA-affected offspring. The reduced expression of genes for the mitochondrially encoded NADH:ubiquinone oxidoreductase core subunit 1 (*mt-Nd1*) and cytochrome C oxidase I (*mt-Co1*), reduced level of protein ND1, reduced activity of respiratory complex I, and reduced MMP pointed to impaired mitochondria as a probable source of ROS. However, electron-microscopic analysis showed morphological alterations only in synaptic mitochondria; mitochondria located elsewhere in neurons or in other cell types were not changed. Obviously, sub-population of synaptic mitochondria is relatively small; therefore, it cannot be responsible for significant changes of MMP or complex I activity (Figure 8). Our data indicated that a significant population of mitochondria is affected in MIA hippocampus; however, swelling occurred specifically in sub-population of synaptic mitochondria. We speculate that the reason of specific synaptic swelling of mitochondria could be higher susceptibility of synaptic mitochondria to stress and/or additional synapse-specific factors enhancing stress in synapses (Fedorovich et al., 2017).

The growing body of evidence indicates the special features of synaptic mitochondria. They suffer alterations during brain ageing and during the pathogenesis of neurodegenerative disorders (Villa et al., 2006; Lores-Arnaiz and Bustamante, 2011; Lores-Arnaiz et al., 2016). Comparing to non-synaptic mitochondria, they possess a special lipid and protein composition (Lai et al., 1977; Rendon and Masmoudi, 1985; Kiebish et al., 2008; Völgyi et al., 2015). Also, the size and activity of crucial mitochondrial enzymes are different in synaptic mitochondria (Kiebish et al., 2008; Fedorovich et al., 2017). Another critical issue may be the cytosolic level of calcium ions in synapses. Mitochondria are involved in calcium uptake and storage. An important role of synaptic mitochondria is to control the calcium level in order to prevent damage during synaptic activity.

Among other factors which could contribute to enhanced stress in synapses, signalling-related processes seem to be the most intriguing. The brain which constitutes ca. 2% of human body weight accounts for about 20% of oxygen consumption. Neurons require huge amounts of ATP to perform functions related to signalling. A significant part of the brain's energy is utilised on synaptic transmission, and the synaptic vesicle cycle is the major consumer of ATP produced by synaptic mitochondria (Harris et al., 2012; Rangaraju et al., 2014). A high level of mitochondrial activity implies an increased ROS generation; therefore, synaptic mitochondria are exposed to bigger oxidative stress than non-synaptic mitochondria. Our research shows much greater disturbance of synaptic mitochondria compared to non-synaptic mitochondria and suggests that the function of synaptic- and non-synaptic mitochondria should be investigated in experimental models and in human ASD clinical studies.

Mitochondrial impairment and oxidative stress have been implicated in neurodevelopmental and neuropsychiatric disorders (Hollis et al., 2017; Roberts, 2017; Joshi and Mochly-Rosen, 2018; Pei and Wallace, 2018). Importantly, valproic acid, which is used as prenatal toxin in experimental models of autism, also inhibits oxidative phosphorylation (OXPHOS) (Haas et al., 1981). Moreover, analysis of transcriptome revealed that genes related to mitochondrial function were expressed differently in autism and schizophrenia, and they correlated with genes related to synaptic function (Arion et al., 2015; Schwede et al., 2018).

The major sites of ROS production in the mitochondrial ETC are complex I and complex III. In our study, we observed a reduced level of mitochondrially encoded mRNA for the *mt-Nd1* gene, followed by a reduced level of NADH:ubiquinone oxidoreductase core subunit 1 (ND1) protein and reduced activity of complex I. Of course, it cannot be excluded that other subunits of complex I could also be affected in the hippocampi of MIA-affected offspring. Altogether, our data suggest that impairment of complex I may be responsible for generation of ROS and may contribute to synaptic dysfunction in animals prenatally exposed to MIA. Our results also indicated that the mRNA level for *mt-Co1* decreased, but neither the protein level of COX1 nor the activity of complex IV changed. Interestingly, the level of mRNA for *mt-Co1* may be specifically regulated by miRNA-181c (Das et al., 2014). Elevated levels of miRNA-181c were shown previously in a valproic acid rat model of autism, as well as in lymphoblasts from ASD patients (Ghahramani Seno et al., 2011; Olde Loohuis et al., 2015). A recent study by Kos and co-workers demonstrated that miRNA-181c regulates spine size and density and affects axonal length (Kos et al., 2016). A binding site for miRNA-181c is also present in *Sod1* and *Sod2* genes; therefore, increased expression of miRNA-181c could hypothetically be responsible for deficiency of Sod1 and Sod2, leading to weakening of antioxidative defence and contributing to oxidative stress (Sticht et al., 2018). However, the complete role of miRNA-181c in the pathomechanism of neurodevelopmental diseases needs further examination (Supplementary Figure 4).

Alternative sources of ROS in the eukaryotic cell could be enzymatic or non-enzymatic reactions occurring in the cytoplasm, lysosomes, peroxisomes, endoplasmic reticulum, or cellular membranes. In our study we focused on cytotoxic mechanisms related to overactive neuroinflammatory signalling which can significantly contribute to elevated ROS levels. Recent findings highlight the role of microglia in the pathomechanism of neurodevelopmental diseases (Takano, 2015; Bergdolt and Dunaevsky, 2019). Our previous study demonstrated that adolescent MIA-offspring showed elevated blood cytokine levels (Cieślik et al., 2020). Moreover, opposite to hippocampus, microglial activation, increased pro-inflammatory cytokines expression, and increased oxidative stress was observed in in the cerebral cortex of adolescent MIA rats (Cieślik et al., 2020). In our analysis of cytokines, the correlation between transcript abundances and protein abundances was weak, which may be explained by a strong regulatory role for processes downstream of transcription (Vogel and Marcotte, 2012). In the case of IFN-γ, an alternative explanation is the impairment of the blood-brain barrier, enabling infiltration of peripheral IFN-γ into the brain (Asakawa and Matsushita, 1980; Simoes et al., 2018) or accelerated transport from blood (Pan et al., 1997). Our results suggest that LPS-induced MIA evokes long-lasting changes in microglia density but not in M1 activation status in the hippocampi of offspring. Only an increase in expression of arginase and IL-10 may indicate activation of the M2a phenotype.

Our results suggest that density, but not cytotoxic activation state of microglia, is changed in MIA-affected offspring. The results of previous studies on microglial activation in rodent MIA models are contradictory. Some studies showed an increase in density and others in activation, and there are also results indicating that microglia is not altered in MIA-affected offspring (Juckel et al., 2011; van Den Eynde et al., 2014; Giovanoli et al., 2016). The effect was also age- and brain structure-specific. However, it is likely that sensitivity of offspring to secondary insults in adult life (stress or inflammation) may be increased (Giovanoli et al., 2013).

Biomarkers of oxidative stress and antioxidant defence in the plasma and brain were shown to be altered in schizophrenia, ASD, and other neurodevelopmental disorders (Smaga et al., 2015). Additionally, our data demonstrated oxidative stress in the hippocampi of MIA-affected offspring. Several studies showed impairment of glutathione system in ASD brain (Bjørklund et al., 2020). It was suggested that significant decrease in the level of cysteine, as the rate-limiting amino acid for glutathione synthesis, may be responsible for impairment of glutathione system and, in consequence, for oxidative stress and mitochondrial dysfunction (James et al., 2006). Alterations of glutathione system and DCF oxidation, which were presented above (Figure 5), indicate oxidative stress in the brain. The consequence of enhanced generation of ROS is oxidative damage to DNA, proteins and lipids, but also modification of several signalling cascades in the cell. Increasing evidence also indicates cross-talk between ROS and calcium homeostasis in the cell (Gorlach et al., 2015). ROS increase cytosolic calcium level, and on the other hand, calcium overload increases ROS generation. Interestingly, mitochondria are at a crossroads of ROS generation and calcium homeostasis; therefore, their impairment may be considered a crucial node of MIA-evoked changes. Additionally, swelling of endoplasmic reticulum, as was shown in electron microscopic analysis, supports the assumption that calcium homeostasis is dysregulated.

Previous studies demonstrated that oxidative stress affects activity of Cdk5 (Lee et al., 2000, 2008; Shea et al., 2004). The mechanism of calcium- and calpain-dependent overactivation of Cdk5 in the brain was observed in neurodegenerative diseases (Wilkaniec et al., 2016), but also during chronic stress (Bavley et al., 2017) and after perinatal exposure to Pb (Ga̧ssowska et al., 2016a). Moreover, the specific mechanism linking overactivation of Cdk5 to hypophosphorylation of MAPT in neuronal cells exposed to oxidative stress was described previously (Zambrano et al., 2004). Oxidative stress-activated Cdk5 executed phosphorylation of I-2 (PP1 inhibitor-2), relieving its inhibitory effect on PP1 (protein phosphatase 1) and leading in consequence to enhanced dephosphorylation of MAPT. Cdk5 is critically important in governing development of the CNS. It controls both neuronal migration and synaptic formation and function. Therefore, dysregulation of its activity might potentially contribute to biochemical and morphological alterations in neurodevelopmental diseases (Lai and Ip, 2015; Ohshima, 2015).

An increased level of p25 protein that is liberated by calcium-activated calpains indicates cytosolic calcium increase and overactivation of Cdk5. Although contribution of the I-2/PP1 pathway cannot be completely excluded, our results suggest that in MIA-affected offspring, other mechanisms may be principal. The enhanced activity of Cdk5 does not evoke hyperphosphorylation of MAPT, but it may be responsible for phosphorylation of the major tau kinase Gsk-3β on Ser9, its inhibition and, in consequence, hypophosphorylation of MAPT. In accordance, as demonstrated above, phosphorylation at Ser199/202 and Ser396 on MAPT was significantly reduced.

The previous data show that MAPT is involved in regulation of synaptic function (Regan et al., 2015; Suzuki and Kimura, 2017; Babur et al., 2019). MAPT is an important protein that binds to microtubules, stabilises them and hence regulates the function of the microtubule-based cytoskeleton. Phosphorylation is involved in intra-axonal sorting of MAPT. Phosphorylated MAPT was localised in soma and dephosphorylated in a growth cone (Mandell and Banker, 1996). Moreover, synaptic activation also evokes phosphorylation-related translocation of MAPT to the synaptic compartment (Frandemiche et al., 2014). However, contrary to axons, only a small amount of MAPT was found in synaptic endings (Mondragon-Rodriguez et al., 2012), where it may interact with several cellular partners (tubulin, F-actin, Src family kinases) and mediate alterations in the structure of synapses (Sotiropoulos et al., 2017). Recent studies demonstrated that MAPT is a binding partner of synapsin (Stefanoska et al., 2018), and dysregulation of MAPT may impact SV mobility and release rate (Moreno et al., 2016; Zhou et al., 2017). The most commonly observed disadvantageous alteration of MAPT in neurons is hyperphosphorylation, which occurs in many pathological conditions, including neurodegenerative disorders, traumatic brain injury, ischemia, epilepsy, and environmental stress (Sotiropoulos et al., 2017). Hyperphosphorylated MAPT dissociates from microtubules, leading to impairment of the cytoskeleton's function and axonal transport. However, Rodriguez-Martin et al. demonstrated that reduced phosphorylation of MAPT may be linked to impairment of transport along the axon and to a decrease in the number of axonal mitochondria. Therefore, it may lead to dysregulation of mitochondrial activity (Rodriguez-Martin et al., 2016). Moreover, Erin Schuman's group showed recently that dendritic mitochondria exist as compartments which are fastened by cytoskeletal tethering (Rangaraju et al., 2019). Because local synthesis of synaptic proteins requires mitochondria-delivered energy, we can expect that disruption of the synaptic cytoskeleton due to impairment of MAPT could affect local synthesis of proteins in synapses, leading in consequence to changes of synaptic structure and function. In accordance, our electron microscopic analysis of mitochondria revealed morphological alterations in nerve endings. Interestingly, mitochondrial and bioenergetic abnormalities were observed in patients with ASD and schizophrenia (Palmieri and Persico, 2010; Sullivan et al., 2018). However, the connexion between MIA-evoked MAPT impairment and mitochondrial dysfunction needs further evaluation.

Another important consequence of dysregulation of MAPT-related processes may be dysfunction of synaptic proteins. MAPT knock-out mice develop the loss of hippocampal excitatory synaptic proteins (Ma et al., 2014). Our TEM analysis of hippocampi revealed important changes in the brains of adolescent offspring rats subjected prenatally to LPS-evoked MIA. These data clearly indicate that synaptic organisation is changed. Moreover, we observed changes in expression and level of synaptic proteins synaptophysin, synapsin, PSD-95, and VAMP1/2. However, other synaptic proteins (syntaxin-1, SNAP-25, and synaptotagmin) were not changed. The MAPT-mediated mechanism responsible for alterations of synaptic protein levels cannot explain alterations of mRNA levels for these proteins.

Synaptophysin is a transmembrane glycoprotein of SVs. Gordon et al. demonstrated that small alterations in synaptophysin level may affect targeting of synaptobrevin to SVs and in consequence may result in decreased fidelity of neurotransmission and synaptic dysfunction (Gordon et al., 2016). Synaptic dysfunction, including changes of synaptic proteins, was shown previously in rats after prenatal activation of the immune system in late gestation by administration of PIC (Oh-Nishi et al., 2010; Forrest et al., 2012). However, Oh-Nishi et al. observed a reduced level of synaptophysin in the hippocampi of MIA-affected offspring (Oh-Nishi et al., 2010). The difference, compared to our results, may be due to a difference in experimental conditions, such as usage of various immune stimulants (PIC vs. LPS) or induction of MIA at various gestation time points (GD15-17 vs. GD9.5). It seems that these factors may significantly alter the environment to which the embryo is exposed, leading to various outcomes in the adolescent brain (Smolders et al., 2018). The changes in the level of synaptophysin did not correlate with changes in the number of SVs; however, the function of SVs may be impaired.

Synapsins are abundant presynaptic proteins involved in synaptogenesis and neuronal plasticity, and phosphorylation of synapsins plays an important role in mediating the trafficking of SVs. Phosphorylation of synapsin I at Ser62/67 by extracellular signal-regulated kinase (ERK1/2) promotes its dissociation from vesicles and, in consequence, dissociation of SVs from the actin cytoskeleton, leading to their mobilisation from the reserve pool to the release-ready pool (Chi et al., 2003). It was shown that calcium regulates the activity of ERK, which could explain the mechanism of increased phosphorylation of this kinase at Ser62/67 (Chuderland and Seger, 2008).

Postsynaptic density protein 95 (PSD-95) encoded by the *DLG4* gene is almost exclusively located in the postsynaptic density of neurons and is involved in anchoring synaptic proteins; for example, it is involved in the recruitment and stabilisation of glutamate receptors (Coley and Gao, 2018). Reduced expression and level of PSD-95 coincided with changes in the ultrastructure of the synaptic cleft, which was observed through electron microscopic analysis, supporting the hypothesis that dysregulation of PSD-95 has an overwhelming impact on synaptic connectivity and activity (Coley and Gao, 2018). Even if some mechanisms may compensate for the deficiency of PSD-95 (Elias et al., 2006), a genetic association of the *DLG4* gene with autism and schizophrenia was demonstrated (Feyder et al., 2010; Purcell et al., 2014).

We observed a lack of correlation between protein abundance and mRNA expression levels, which is commonly reported in many biological systems (Greenbaum et al., 2003). One can only speculate on the reason. The first explanation of this phenomenon is that various post-transcriptional mechanisms involved in turning mRNA into protein may be affected in MIA-affected offspring; second, proteins may significantly differ in their half-lives, which is the result of synthesis and degradation. Interestingly, alterations of protein synthesis and degradation are observed in neurodevelopmental disorders. For example, in ASD, increased synthesis and reduced degradation *via* autophagy were observed as the result of overactivation of kinase mTOR (Santini et al., 2013; Tang et al., 2014; Sato, 2016). Our preliminary results demonstrated that due to enhanced phosphorylation, activity of mTOR kinase may be increased in the hippocampi of MIA-affected offspring, leading in consequence to impairment of protein synthesis and degradation (Supplementary Figure 2B).

The limitation of our study, which should be avoided in further research, is that the experiments were performed on male young-adult offspring. The impact of gender on key MIA-evoked changes presented in this study should be analysed in detail in future research. Other unanswered question is, why functional analysis of mitochondria indicated enormous pathology, whereas electron microscopic analysis showed morphological changes of mitochondria only within synapses. We suppose that even if ultrastructural changes were limited to nerve endings, it cannot be excluded that function of mitochondria is affected also in other parts of neuronal cells, or even in other cell types. Therefore, because of heterogeneity of mitochondrial population, it would be reasonable to perform functional analysis on separate mitochondrial populations: synaptic vs. non-synaptic. Moreover, because the density of microglia seems to be increased in MIA-offspring, it would be important for the future studies to analyse biochemical/molecular processes and mitochondrial function separately in various cell types, especially in microglial cells. The future studies should also include analysis of the physiological and functional neuronal changes, as a consequence of the morphological and molecular alterations.

Overall, our results demonstrated the variety of molecular alterations in the hippocampi of male adolescent rats exposed to inflammatory insult during prenatal life. Despite the hippocampus being especially sensitive to inflammatory insults, it seems that inflammatory processes, including microglial activation, are not increased. The impairment of the mitochondrial function, enhanced generation of ROS, activation of Cdk5 and inactivation of Gsk-3β followed by hypophosphorylation of MAPT potentially could contribute to dysregulation of synapses. However, we cannot rule out that these pathological changes are secondary to synaptic dysfunction. Our results, for the first time, clearly reveal that MIA in early gestation contributes to aberration of the synaptic ultrastructure in the hippocampi of adolescent offspring, which may be responsible for disturbances in the cell signalling pathway, synaptic plasticity and cognitive skills. While illuminating the complexity of the interaction of immune response in MIA with oxidative stress, mitochondria dysfunction and synapses condition in the adolescent offspring brain, our results may be especially important in revealing the currently under-emphasised influence of maternal bacterial infection on the adolescent offspring response to MIA.

## Data Availability Statement

The raw data supporting the conclusions of this article will be made available by the authors, without undue reservation.

## Ethics Statement

The animal study was reviewed and approved by Local Ethics Committee for Animal Experimentation in Warsaw.

## Author Contributions

AA, MC, GC, and MG-D conceived and designed the experiments. MC, GC, MG-D, AZ, MF-B, MG, and AD performed the experiments. MC, GC, MG-D, MF-B, MG, and AA analysed and interpreted the data. MC and AA contributed reagents, materials, analysis tools. GC, MC, and AA wrote the paper. All authors read and approved the final manuscript.

## Conflict of Interest

The authors declare that the research was conducted in the absence of any commercial or financial relationships that could be construed as a potential conflict of interest.
