# Supplementary material for: The Synaptic Dysregulation in Adolescent Rats Exposed to Maternal Immune Activation
Source: Front Mol Neurosci. 2021 Jan 14;13:555290. doi: 10.3389/fnmol.2020.555290 (PMC7840660; doi:10.3389/fnmol.2020.555290)
Supplement: Supplementary file 1 [file Data_Sheet_1.pdf]

Supplementary Material

1 List of key materials

1.1 TaqMan Gene expression Assays

Actb	Rn 00667869_m1	Thermo Fisher Scientific
Cd86	Rn00571654_m1	Thermo Fisher Scientific
Chi3l1	Rn01490608_m1	Thermo Fisher Scientific
Dlg4	Rn00571479_m1	Thermo Fisher Scientific
Fcgr1a	Rn01762682_m1	Thermo Fisher Scientific
Ifng	Rn00594078_m1	Thermo Fisher Scientific
Il10	Rn99999012_m1	Thermo Fisher Scientific
Il1b	Rn00580432_m1	Thermo Fisher Scientific
Il6	Rn01410330_m1	Thermo Fisher Scientific
miR-181c-5p	Rno-miR-181c-5p	Thermo Fisher Scientific
miR-191	Rno-miR-191a-5p	Thermo Fisher Scientific
Mrc1	Rn01487342_m1	Thermo Fisher Scientific
mt-Co1	Rn03296721_s1	Thermo Fisher Scientific
mt-Cytb	Rn03296746_s1	Thermo Fisher Scientific
mt-Nd1	Rn03296764_s1	Thermo Fisher Scientific
Sdha	Rn00590475_m1	Thermo Fisher Scientific
Sod1	Rn00566938_m1	Thermo Fisher Scientific
Sod2	Rn00566942_g1	Thermo Fisher Scientific
Sphk1	Rn00591307_m1	Thermo Fisher Scientific
Syn1	Rn00569468_m1	Thermo Fisher Scientific
Syp	Rn01528256_m1	Thermo Fisher Scientific
Syt1	Rn 00436862_m1	Thermo Fisher Scientific
Tgfb1	Rn00572010_m1	Thermo Fisher Scientific
Tnf	Rn99999017_m1	Thermo Fisher Scientific
Vamp1	Rn00565308_m1	Thermo Fisher Scientific
Vamp2	Rn00360268_g1	Thermo Fisher Scientific

1.2 Antibodies -Western blotting

donkey anti-goat IgG	sc-2056	Santa Cruz Biotechnology
goat anti-rabbit IgG	A0545	Sigma-Aldrich
goat polyclonal anti-Iba1	ab5076	Abcam
mouse monoclonal anti-MAPT	sc-32274	Santa Cruz Biotechnology
mouse monoclonal anti-nitrotyrosine	sc-32757	Santa Cruz Biotechnology
mouse monoclonal anti-phospho-MAPT(S396)	#9632	Cell Signaling
mouse monoclonal anti-PSD-95	sc-71935	Santa Cruz Biotechnology
mouse monoclonal anti-synapsin	sc-390867	Santa Cruz Biotechnology
mouse monoclonal anti-synaptophysin	sc-55507	Santa Cruz Biotechnology
mouse monoclonal anti-VAMP1/2	sc-20039	Santa Cruz Biotechnology
mouse monoclonal anti-arginase	sc-166920	Santa Cruz Biotechnology

mouse monoclonal anti-MTCO1	ab14705	Abcam
mouse monoclonal anti-ND1	sc-293243	Santa Cruz Biotechnology
mouse monoclonal anti-s100b	S2532	Sigma-Aldrich
mouse monoclonal anti-syntaxin-1	sc-12736	Santa Cruz Biotechnology
rabbit polyclonal anti-GAPDH	G9545	Sigma-Aldrich
rabbit monoclonal anti-Gsk-3β	#9315	Cell Signaling
rabbit monoclonal anti-phospho-MAPT(S416)	#15013S	Cell Signaling
rabbit monoclonal anti-mTOR	#2983	Cell Signaling
rabbit monoclonal anti-phospho-mTOR(S2448)	#5536	Cell Signaling
rabbit monoclonal anti-SNAP25	#5309	Cell Signaling
rabbit monoclonal anti p38	#8690	Cell Signaling
rabbit monoclonal anti phospho-p38(T180/Y182)	#8690	Cell Signaling
rabbit polyclonal anti-Cdk5	sc-173	Santa Cruz Biotechnology
rabbit polyclonal anti-p35/p25	sc-820	Santa Cruz Biotechnology
rabbit polyclonal anti-phospho-Gsk-3β(S9)	#9336	Cell Signaling
rabbit polyclonal anti-phospho-Gsk-3β (S389)	14850-1-AP	Proteintech
rabbit polyclonal anti-phospho-MAPT(S199/202)	T6819	Sigma-Aldrich
rabbit polyclonal anti-phospho-synapsin(S62/67)	sc-135709	Santa Cruz Biotech.
rabbit polyclonal anti-synaptotagmin-1	#3347	Cell Signaling
rabbit polyclonal anti-iNOS	sc-649	Santa Cruz Biotechnology
rabbit polyclonal anti-vinculin	#4650	Cell Signaling
sheep anti-mouse IgG	NXA931V	GE Healthcare

1.3 Antibodies – immunohistochemistry

rabbit polyclonal anti-IL-1 beta	ab9722	Abcam
rabbit polyclonal anti-liver Arginase	ab91279	Abcam
goat polyclonal anti-Iba1	ab5076	Abcam
goat polyclonal anti-GFAP	ab53554	Abcam
donkey polyclonal anti-Goat (Alexa Fluor 594)	A-11058	Thermo Fisher Sci.
donkey polyclonal anti-Rabbit (Alexa Fluor 488)	A-21206	Thermo Fisher Sci.

1.4 Key reagents

LPS (from E. coli serotype 055:B5; Lot 113M4068V)	L2880	Sigma-Aldrich
TRI	T9424	Sigma-Aldrich
DNAse I	AMPD1	Sigma-Aldrich
High Capacity cDNA Reverse Transcription Kit	4374966	Thermo Fisher Sci.
Micro RNA purification kit	035-25	A&A Biotechnology
TaqMan Advanced miRNA cDNA Synthesis Kit	A28007	Thermo Fisher Sci.
TaqMan Fast Advanced Master Mix	4444964	Thermo Fisher Sci.
Bio-Plex Pro Rat Cytokine 23-Plex Assay	12005641	Bio-Rad Laboratories
DCFH-DA	D6883	Sigma-Aldrich

1.5 Commercial Assay Kits

Glutathione Assay Kit	703002	Cayman Chemical Company

Complex I Enzyme Activity Microplate Assay Kit	ab109721	Abcam
Complex IV Rodent Enzyme Activity Microplate Assay Kit	ab109911	Abcam
Mitochondria Isolation Kit	MITOISO1	Sigma-Aldrich

2 Supplementary figures

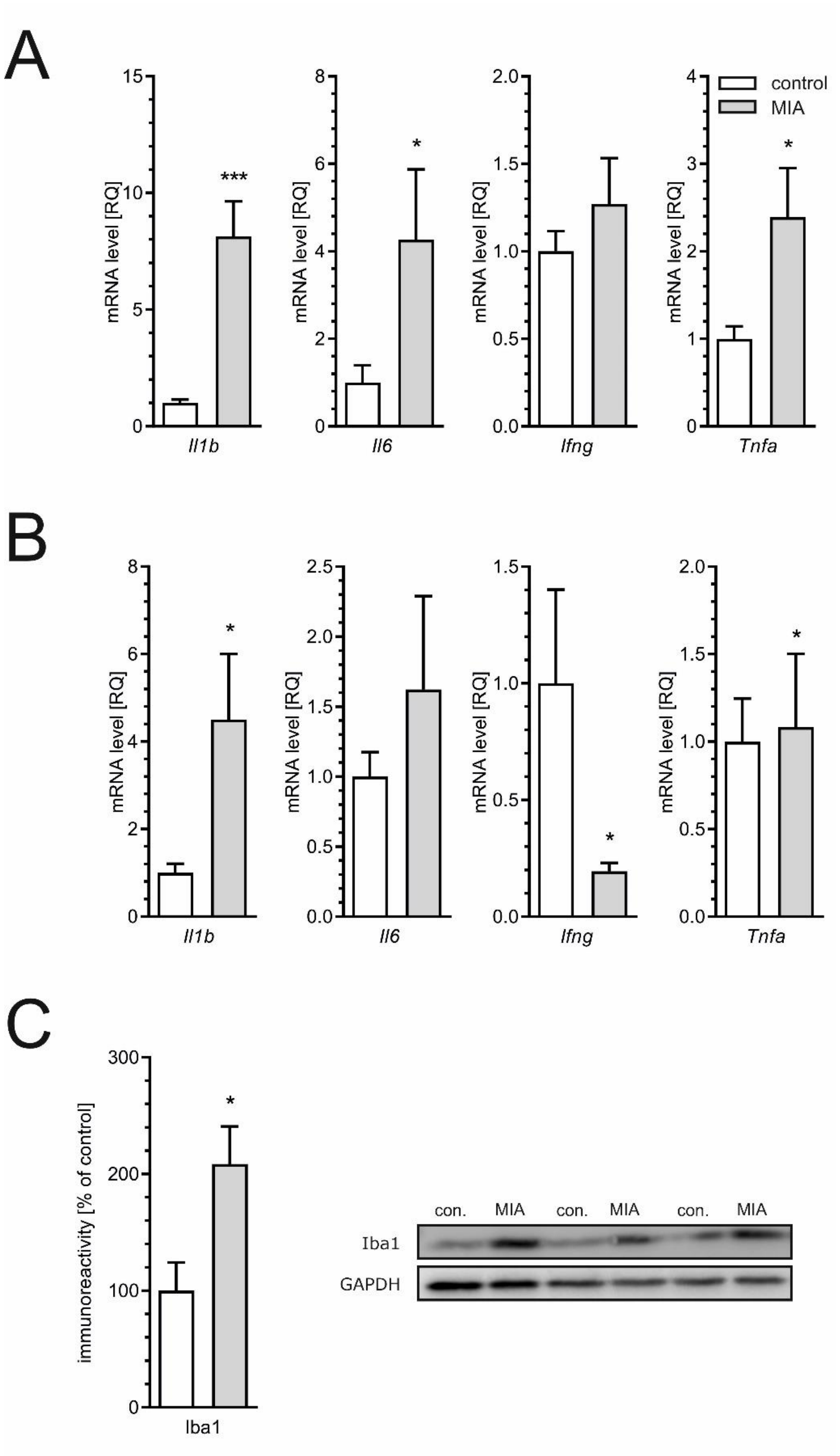

Supplementary Figure 1. Intraperitoneal administration of LPS to pregnant dams stimulates an increase of inflammatory markers in fetuses and placenta. LPS (100 µg/kg b.w.) was injected

intraperitoneally at gestation day 9.5 to female rats. After 24 h rats were decapitated, and the fetuses and placenta were collected. A) The level of mRNA for *Il1b* (n=8), *Il6* (n=7 and 8), *Ifng* (n=8) and *Tnfa* (n=8) in fetuses. B) The level of mRNA for *Il1b* (n=7 and 8), *Il6* (n=7 and 5), *Ifng* (n=7 and6) and *Tnfa* (n=8 and 6) in placenta. C) Densitometric analysis of immunoreactivity of Iba1 (n = 6) in fetuses. Representative immunoblots of Iba1 and GAPDH as a loading control. Each sample presented on representative pictures is from separate animal. * $p<0.05$, *** $p<0.001$, compared with the control group.

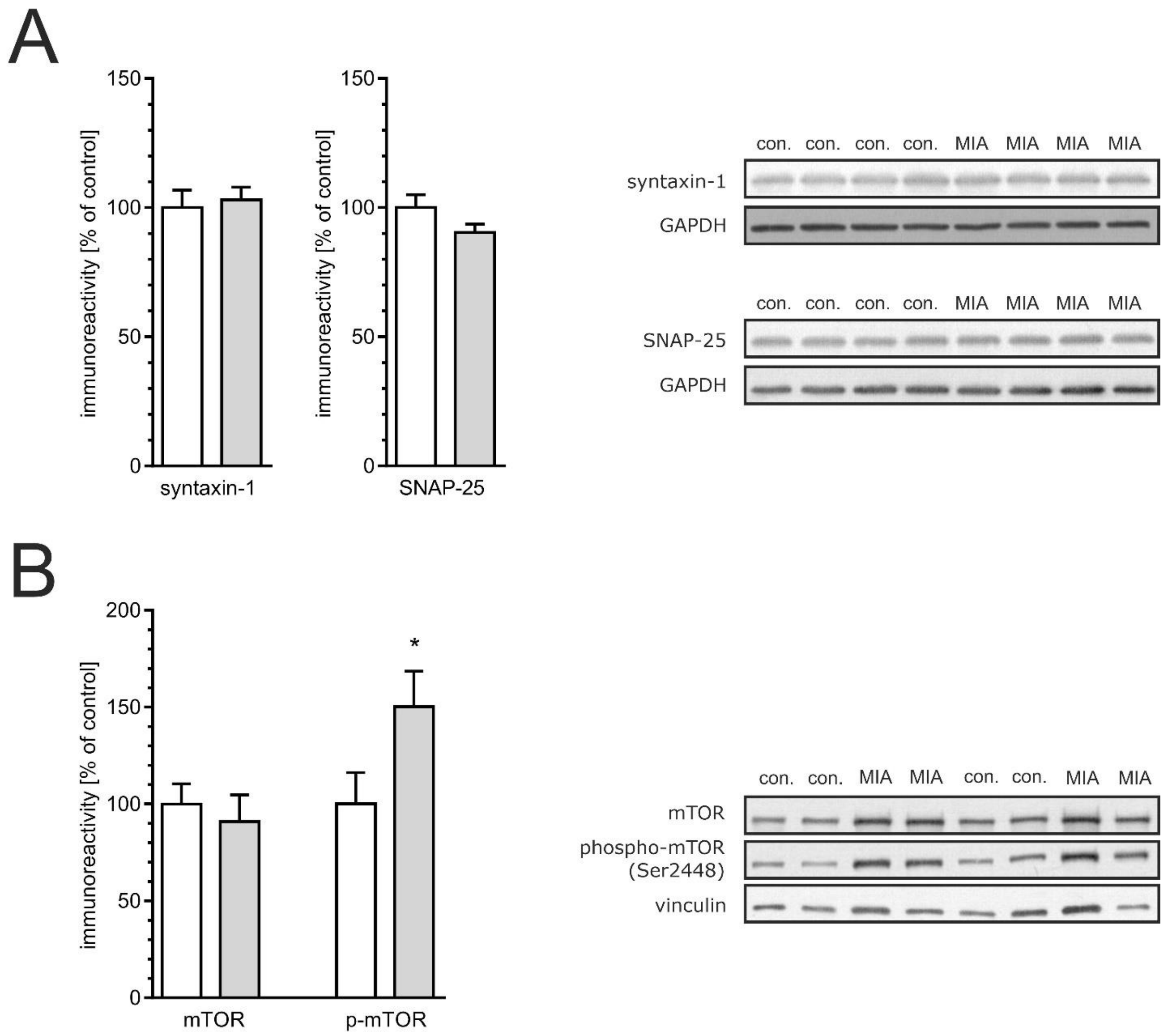

Supplementary Figure 2. Effect of maternal immune activation on immunoreactivity of syntaxin-1, SNAP-25 and mTOR/phospho-mTOR in hippocampi of adolescent male rats. LPS (100 µg/kg b.w.) was injected intraperitoneally at gestation day 9.5 to female rats. Offspring male rats at day 52 were decapitated, and the hippocampi were collected. A) Densitometric analysis of immunoreactivity of syntaxin-1 (n = 5) and SNAP-25 (n = 5). Data were normalized to GAPDH immunoreactivity. Representative immunoblots are presented. B) Densitometric analysis of immunoreactivity of mTOR (n= 6 and 5) and p-mTOR (Ser2448) (n = 5 and 6). Data were normalized to vinculin immunoreactivity. Representative immunoblots are presented. * $p < 0.05$, compared with the control group.

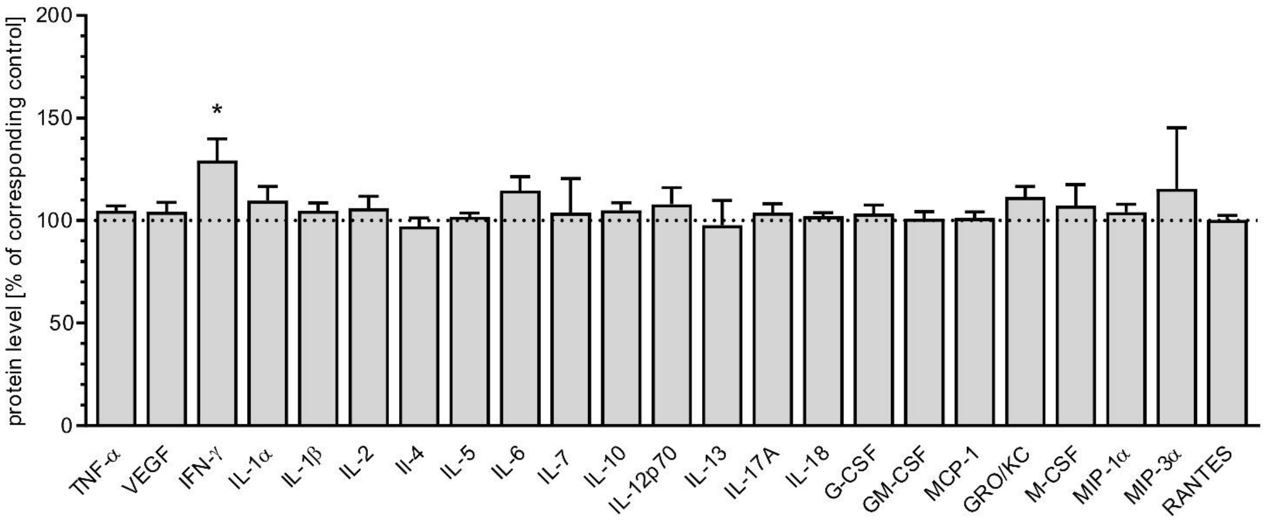

Supplementary Figure 3. Effect of maternal immune activation on levels of inflammatory mediators in hippocampi of adolescent male rats. The level of cytokines in hippocampal extract from adult male rats was determined by using the Bio-Plex Pro Rat Cytokine 23-Plex Assay on the Luminex Bio-Plex 200 system (n = 7). Presented results are relative changes, compared to control rats. * $p < 0.05$, compared with the control group.

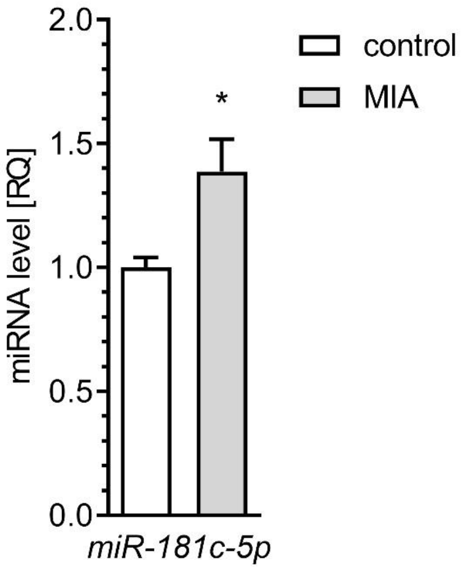

Supplementary Figure 4. Maternal immune activation increases the level of micro RNA miR-181c-5p in hippocampi of adolescent male rats. LPS (100 µg/kg b.w.) was injected intraperitoneally at gestation day 9.5 to female rats. Offspring male rats at day 52 were decapitated, and the hippocampi were collected. The level of micro RNA was measured by real-time PCR and calculated by the ΔΔCt method with miR-191 as a reference gene (n = 8 and 9).

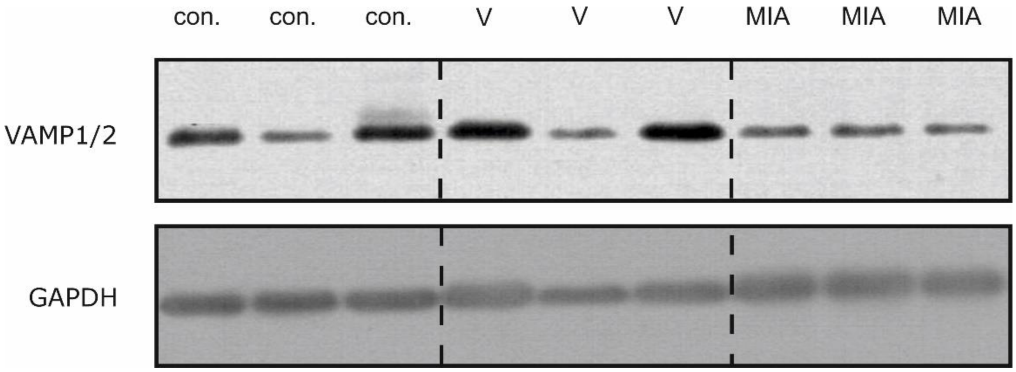

Supplementary figure 5. Full scans of the entire original immunoblots presented in re-arranged form on Figure 3. Three redundant lanes in the middle (V) representing other experimental group, not related with the current study, were removed.
